# *EXOSC8* mutations alter mRNA metabolism and cause hypomyelination with spinal muscular atrophy and cerebellar hypoplasia

**DOI:** 10.1038/ncomms5287

**Published:** 2014-07-03

**Authors:** Veronika Boczonadi, Juliane S. Müller, Angela Pyle, Jennifer Munkley, Talya Dor, Jade Quartararo, Ileana Ferrero, Veronika Karcagi, Michele Giunta, Tuomo Polvikoski, Daniel Birchall, Agota Princzinger, Yuval Cinnamon, Susanne Lützkendorf, Henriett Piko, Mojgan Reza, Laura Florez, Mauro Santibanez-Koref, Helen Griffin, Markus Schuelke, Orly Elpeleg, Luba Kalaydjieva, Hanns Lochmüller, David J. Elliott, Patrick F. Chinnery, Shimon Edvardson, Rita Horvath

**Affiliations:** 1 Institute of Genetic Medicine, Wellcome Trust Centre for Mitochondrial Research, Newcastle University, Central Parkway, Newcastle upon Tyne NE1 3BZ, UK; 2 The Monique and Jacques Roboh Department of Genetic Research, Hadassah– Hebrew University Medical Center, Jerusalem 91120, Israel; 3 Department of Life Sciences, University of Parma, Parco Area delle Scienze 11A, Parma 43124, Italy; 4 Department of Molecular Genetics and Diagnostics, NIEH, Albert Florian ut 2-6, Budapest 1097, Hungary; 5 Department of Pathology, Institute for Ageing and Health, Newcastle University, Campus for Ageing and Vitality, Newcastle upon Tyne NE4 5PL, UK; 6 Neuroradiology Department, Regional Neurosciences Centre, Queen Victoria Road, Newcastle upon Tyne NE1 4PL, UK; 7 Department of Paediatrics, Josa Andras Hospital, Szent Istvan utca 6, Nyiregyhaza 4400, Hungary; 8 Department of Poultry and Aquaculture Sciences, Institute of Animal Science, Agricultural Research Organization, The Volcani Center, P.O.Box 6, Bet Dagan 50250, Israel; 9 Department of Neuropediatrics and NeuroCure Clinical Research Center, Charité-Universitätsmedizin, Charité-Platz 1, 10117 Berlin, Germany; 10 Western Australian Institute for Medical Research/Centre for Medical Research, The University of Western Australia, 35 Stirling Highway Crawley, Western Australia 6009 Perth, Australia; 11 These authors contributed equally to this work

## Abstract

The exosome is a multi-protein complex, required for the degradation of AU-rich element (ARE) containing messenger RNAs (mRNAs). EXOSC8 is an essential protein of the exosome core, as its depletion causes a severe growth defect in yeast. Here we show that homozygous missense mutations in *EXOSC8* cause progressive and lethal neurological disease in 22 infants from three independent pedigrees. Affected individuals have cerebellar and corpus callosum hypoplasia, abnormal myelination of the central nervous system or spinal motor neuron disease. Experimental downregulation of *EXOSC8* in human oligodendroglia cells and in zebrafish induce a specific increase in ARE mRNAs encoding myelin proteins, showing that the imbalanced supply of myelin proteins causes the disruption of myelin, and explaining the clinical presentation. These findings show the central role of the exosomal pathway in neurodegenerative disease.

## Abstract (web summary)

The exosome is responsible for mRNA degradation, which is an important step in the regulation of gene expression. Here the authors report that homozygous missense mutations in the exosome subunit, EXOSC8, may cause neurodegenerative disease in infants through the dysregulation of myelin expression.

## Graphical abstract

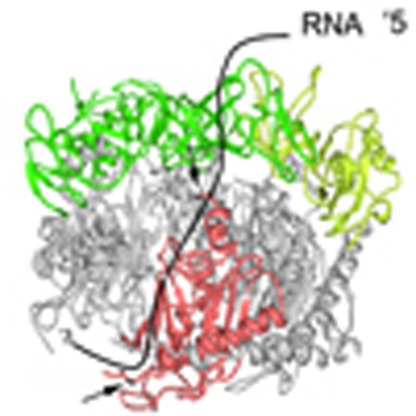

The degradation of messenger RNAs (mRNAs) is an important regulatory step, which controls gene expression^1^,^2^. Unstable mammalian mRNAs contain AU-rich elements (AREs) within their 3′-untranslated regions. Rapid degradation of ARE-containing RNAs is performed by a multi-protein complex, the exosome^3^,^4^. The versatility and specificity of the exosome regulate the activity and maintain the fidelity of gene expression^5^. Both in yeast and humans, nine proteins organize the ‘exosome core’ in a two-layered ring. The central hexamer channel is composed by six subunits (Rrp41p/EXOSC4, Rrp46p/EXOSC5, Rrp45p/PM/Scl-75/EXOSC9, Rrp42p/EXOSC7, Mtr3/EXOSC6 and Rrp43p/Oip2/EXOSC8), while the cap consists of three proteins (Rrp4/EXOSC2, Rrp40/EXOSC3 and Csl4/SOCS4)^4^,^6^. The exosome degrades RNA starting at the 3′-end by an exoribonucleolytic function and it also has an endoribonucleolytic function^7^,^8^. The catalytic activity of the exosome core is provided through the association with other proteins (RRP44/DIS3, RRP6/PM/Scl-100/EXOSC10 ribonucleases)^1^. ARE recognition requires ARE-binding proteins that interact with the exosome for recruitment, thereby promoting the rapid degradation of target RNAs^9^.

The human exosome also regulates gene expression via diverse RNA processing reactions^10^. Many cellular RNAs that play key roles in important cellular processes such as translation (ribosomal RNAs, transfer RNAs and small nucleolar RNAs) and mRNA splicing (small nuclear RNAs) are produced as precursor molecules that are trimmed from their 3′-ends by the human exosome^11^. This complex organization of the exosome provides the versatility needed to cope with the huge variety of RNA substrates in the cell^12^. However, detailed *in vivo* analyses are technically challenging and many questions remain unresolved.

Here we report that deficiency of a core component of the human exosome leads to severe infantile overlap phenotype of psychomotor deficit, cerebellar and corpus callosum hypoplasia, hypomyelination and spinal muscular atrophy (SMA).

## Results

### Clinical presentation in 22 patients from three pedigrees

**Figure 1:**
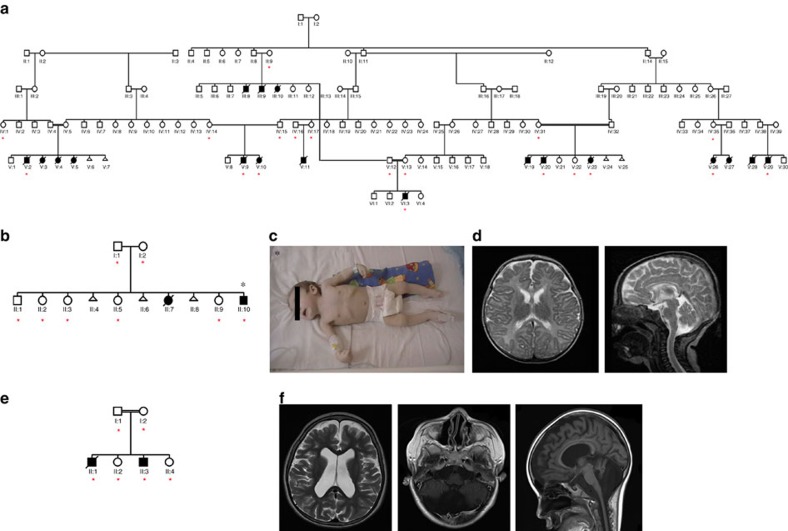
Pedigrees with clinical presentation and brain MRI. (**a**) Pedigree of the original Hungarian Roma family. *DNA of these family members was used for mutation analysis. (**b**) Pedigree 2, Hungarian Roma ethnic origin. (**c**) Patient P-II:10 at age 6 months. (**d**) MRI of patient P-II:10-detected immature myelination, which was consistent with the patient’s age (5 months, axial T2 image) A moderately thin corpus callosum was seen in the sagittal T2 image of the same patient. The extra-cerebral cerebrospinal fluid spaces were satisfactory in appearance. (**e**) Pedigree 3, a consanguineous Palestinian family. (**f**) Brain MRI of the affected siblings revealed mega cisterna magna and hypoplasia of the cerebellum and corpus callosum.

First we studied a large Hungarian family of Roma ethnic origin, where 18 children presented between 2–4 months of age with failure to thrive, severe muscle weakness, spasticity and psychomotor retardation. Vision and hearing were impaired in all patients and deterioration was usually triggered by inter-current infections. All affected children died of respiratory failure before 20 months of age (Fig. 1a, Supplementary Table 1). Detailed diagnostic workup excluded known metabolic, neurodegenerative and common genetic disorders. Electrophysiology was performed in one patient only (P1-V:10) and was not conclusive. Brain magnetic resonance imaging (MRI) showed variable abnormalities including vermis hypoplasia, immature myelination, cortical atrophy and thin corpus callosum (Supplementary Table 1).

*EXOSC8* mutations were also identified in two additional patients from an independent Hungarian Roma family (Fig. 1b,c) and in two affected siblings from a consanguineous Arab–Palestinian family (Fig. 1e). The clinical presentation of the additional patients was compatible with a progressive, infantile onset neurological disease, presenting with severe muscle weakness, respiratory problems, developmental delay and early death (Supplementary Table 1). Vermis hypoplasia was more prominent in the third pedigree (Fig. 1f), while immature myelination was reported in pedigree 2 (Fig. 1d). Weakness in P3 was proximal more than distal with attendant tongue fasciculations. Motor neuronopathy was noted on electrophysiological examination in P3-II:1.

Muscle biopsy in patient P1-V:10 at 5 months of age detected variations in fibre size and increased subsarcolemmal nuclei, but no signs of SMA. Cytochrome *c* oxidase (COX) negative fibres were noted on histochemical staining and activities of respiratory chain complexes I and IV were moderately decreased (Supplementary Note 1). We excluded mitochondrial DNA (mtDNA)-mediated mechanisms for multiple respiratory chain enzyme deficiency (mtDNA deletion/depletion, point mutations). Muscle biopsy of another patient (P3-II:1) at 2 years of age showed groups of atrophic and hypertrophic fibres compatible with SMA.

**Figure 2:**
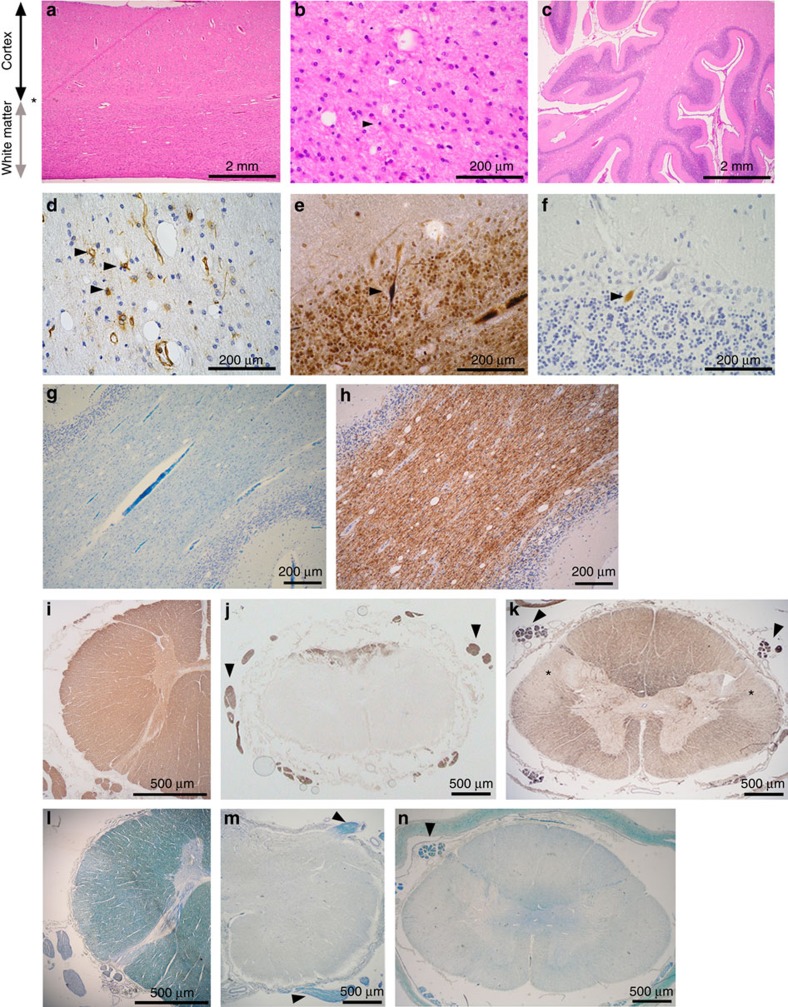
Autopsy findings. (**a**–**h**) Cerebral cortex and white matter in autopsy of patient V:3 (pedigree 1). (**a**,**b**) Thickness of the white matter is significantly reduced, while the cortex is relatively well preserved. Black arrow: thickness of the cortex from the pia to the cortex/white matter borderzone; grey arrow: thickness of the white matter from the borderzone to the ependymal lining (Hematoxylin and eosin (H&E) staining); extensive astrogliosis (pale nuclei; white arrowhead) and reduced frequency of oligodendrocytes (dark round nuclei; black arrowhead). (**c**) Cerebellar cortex is relatively well preserved, but there is a microvacuolation of the underlying white matter within the cerebellar folia (H&E staining). (**d**) Reactive astrocytes (arrows) within the cerebellar white matter (vimentin immunohistochemistry). (**e**,**f**) Purkinje cell axonal torpedos (modified Bielschowsky silver method) (**e**) and neurofilament antibodies (**f**) indicating the loss of axonal connections. (**g**) Loss of myelin within the cerebellar white matter (Luxol Fast Blue). (**h**) Relative preservation of the myelin staining on myelin basic protein immunohistochemistry. (**i**–**n**) Spinal cord: normal control (**i**,**l**), Pelizaeus–Merzbacher disease (**j**,**m**) and EXOSC8 deficiency (patient V:20) (**k**,**n**). In EXOSC8 deficiency, myelin basic protein is present–apart from the longitudinal descending fibre tracts (**k**, *)–while it is completely absent in Pelizaeus–Merzbacher disease (**j**). Myelin is well preserved within the peripheral nerve roots (**m**,**n**, arrowheads), while indicates severe loss of myelin in both conditions within the spinal cord (**m**,**n**, Luxol Fast Blue).

Autopsy in eight patients detected profound lack of myelin in the cerebral and cerebellar white matter and in the spinal cord, predominantly affecting the descending lateral fibre tracts, while myelination was normal in the peripheral nerves (Fig. 2a–n). The lack of myelin in the brain and spinal cord (Fig. 2g,n) was similarly severe as observed in a patient with Pelizaeus–Merzbacher disease (PMD) (Fig. 2m). However myelin basic protein (MBP) staining was stronger in our patients (Fig. 2k) as compared with the PMD patient (Fig. 2j), indicating the different composition of defective myelin. Autopsy was not performed in the deceased patient from P3-II:1, but MRI at 5 years of age did not indicate hypomyelination.

### Homozygosity mapping and exome sequencing

**Figure 3:**
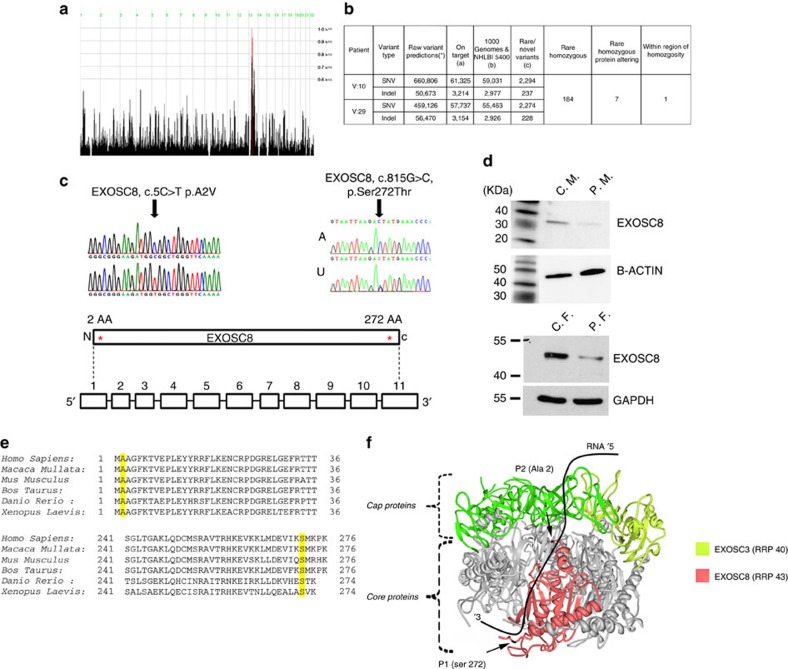
Homozygosity mapping, exome sequencing, *EXOSC8* mutation analysis and immunoblotting for EXOSC8. (**a**) Genome-Wide Human SNP Array 6.0 (Affymetrix) was performed in six affected (P1-V:2, P1-V:10, P1-V:20, P1-V:27, P1-V:29, P1-VI:3) and one unaffected family members (P1-IV:14). Homozygosity mapping indicated two regions of homozygosity on chromosome 13 spanning from 36214563, bp to 37767059, bp (rs7327540 to rs11147637) and 43243791, bp to 44640995, bp (rs9533208 to rs9567354). (**b**) Exome sequencing detected 2,294 and 2,274 rare/novel single nucleotide variants (SNV) of which 184 were shared homozygous variants in patients P1-V:10 and P1-V:29; seven were protein altering, one of which was in the region of homozygosity in *EXOSC8*. SNV-Varscan parameters (*) minimum total coverage≥fivefold, minimum variant coverage ≥onefold, minimum quality>10; Indel-Dindel output filter minimum variant coverage ≥4. (**a**) Variants with position within targets (Illumina Truseq 62 Mb) ±500 bp, seen on both (forward and reverse) strands and (SBVs only) variant allele frequency >24%. (**b**) Variants that match 1,000 Genomes (Feb2012) and/or NHLBI 6500 exomes and/or In-house 334 exomes with MAF>0.01. (**c**) Confirmatory Sanger Sequencing indicated the presence of the homozygous c.815 G>C, p.Ser272Thr mutation in an affected patient (A) and heterozygously in her mother (U). This rare variant is listed as rs36027220 with a minor allele frequency of <0.01 (ref database) and was present also in pedigree 2. In pedigree 3 another homozygous mutation c.5C>T, p.Ala2Val has been identified (control - upper chromatogram, patient - lower chromatogram). (**d**) Immunoblotting showing EXOSC8 protein in control myoblasts (C. M.) and in myoblasts of P1-V:10 (P. M.); control fibroblasts (C. F.) and in fibroblasts of P3-II:1 (P. F.) using β-Actin or GAPDH as a loading control. (**e**) Both mutations alter conserved amino acids. (**f**) Crystal structure of the human exosome complex. The cap proteins are indicated in green (EXOSC3 light green) and the core proteins in grey. EXOSC8 highlighted in pink and the mutated residues (Ser 272, Ala2) are marked as black (arrowhead).

Genome-Wide Human SNP Array 6.0 (Affymetrix) in six affected family members of the first pedigree revealed two broad regions of homozygosity on chromosome 13 (36212278–37767059, bp, size: ~1.55 Mb; and 43243791-44640995, bp, size: 1.4 Mb) (Fig. 3a)^13^. Exome sequencing in genomic DNA of two patients (P1-V:10, P1-V:29) identified seven shared rare (major allele frequency <0.01) homozygous protein altering variants (Fig. 3b), but only one, the c.815G>C, p.Ser272Thr mutation in *EXOSC8* (Gene ID:11340, NC_000013.10, mRNA:NM_181503.2) (Fig. 3d) was located in the larger ~1.55 Mb homozygous region. This mutation affects a conserved amino acid and resulted in significantly decreased EXOSC8 protein (Fig. 3e–g), and no other variants were found in complementary DNA (cDNA) of *EXOSC8*. The location of the mutation implies that it may affect the opening of the RNA channel within the exosome, where funneling of RNAs takes place in single-stranded conformation by an unwinding pore (Fig. 3f). This mutation segregated with the disease in 19 individuals within the family (8 affected: homozygous; 11 unaffected: heterozygous or wild type) (Fig. 1a). The same homozygous mutation was identified in two siblings with an identical clinical presentation from a second Hungarian Roma family (pedigree 2) from the same region. None of the other six homozygous mutations detected by exome sequencing of the index patients in pedigree 1 were homozygous in the patients in pedigree 2, thus excluding the possibility that these other mutations were causing the disease.

To estimate the frequency of this mutation in Roma population, mutation testing was carried out in 63 anonymized Roma controls from Bulgaria, of whom 33 were homozygous for an identical 229522, bp chromosome 13q13.1 haplotype (rs582091 to rs7327020) around c.815G>C, p.Ser272Thr (Supplementary Note 2). This screening identified two heterozygotes, that is, carrier rates of ~3% in the general Roma population, which is in agreement with the frequency rates of other Roma disease causing mutations^14^,^15^.

A different missense mutation, c.5C>T, p.Ala2Val was identified on exome sequencing in two affected siblings of a consanguineous Palestinian family (pedigree 3) with SMA, cerebellar and corpus callosum hypoplasia (Fig. 3c). Homozygosity mapping in this pedigree resulted in the identification of three disease-linked genomic regions, one of them was an 8.4 Mb spanning chr13:31642481-40039652 (Hg19) (rs7996548 to rs12873765). Exome sequencing in genomic DNA of P3:II-3 identified four shared rare (minor allele frequency<0.01) homozygous protein altering variants but only one of them affected an evolutionary conserved residue (Fig. 3e). This variant, c.5C>T, p.Ala2Val (chr13: 37574947 C>T) in the *EXOSC8* gene segregated with the disease in the family, was not present in dbSNP138 and was also absent from the 6503 exome analyses available at the Exome Variant Server, NHLBI GO Exome Sequencing Project (ESP), Seattle, WA, USA (URL: http://evs.gs.washington.edu/EVS/) (accessed on 26 January 2014) and in fibroblasts of the patient, immunoblotting detected severely decreased EXOSC8 protein (Fig. 3d). The c.5C>T mutation in the second codon of *EXOSC8* might therefore either interfere with the Kozak consensus sequence and/or cause mRNA instability probably through interference with the 5′ mRNA capping mechanism^16^,^17^. Nonsense-mediated messenger decay could be excluded because mutant copy numbers could not be increased after 300 mM puromycin treatment (Supplementary Fig. 1).

### Downregulation of the yeast ortholog of human *EXOSC8*

**Figure 4:**
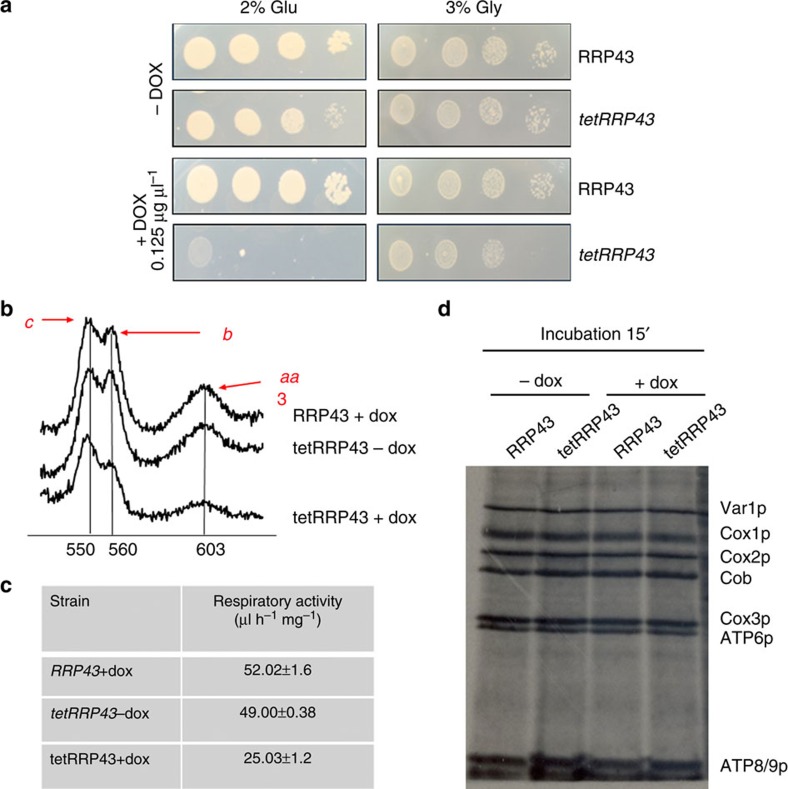
Characterization of the yeast *RRP43* and *tetRRP43* strains to access mitochondrial function. (**a**) Oxidative growth phenotype of *RRP43* and *tetRRP43* strains grown in the presence and absence of doxycycline (0.125 μg ml^−1^). Equal amounts of serial dilutions (10^5^, 10^4^, 10^3^, 10^2^) of cells from exponentially grown cultures were spotted onto YP plates supplemented with 2% glucose (left panel) or with 3% glycerol (right panel). The growth was scored after 3 days of incubation at 28 °C. (**b**) Cytochrome spectra of *RRP43* and *tetRRP43* strains grown in the presence and absence of doxycycline (0.125 μg ml^−1^). The peaks at 550, 560 and 602 nm (vertical bars) correspond to cytochromes *c*, *b* and *aa*_3_, respectively. The height of each peak relative to the baseline of each spectrum is an index of cytochrome content. (**c**) Respiratory activity of yeast *RRP43* and *tetRRP43* strains grown in the absence and in the presence of doxycycline (0.125 μg ml^−1^). Wild-type *RRP43* and *tetRRP43* mutant strain were grown in YP medium supplemented with 0.6% glucose. Respiratory rates were normalized to the wild-type strain grown in the presence of doxycycline. (**d**) Mitochondrial protein synthesis was performed on *RRP43* and *tetRRP43* strains in the presence and absence of doxycycline (0,125 μg ml^−1^), after 15 min of incubation with ^35^S. Equivalent amounts of protein were separated by SDS–PAGE on a 17.5% polyacrylamide gel, transferred to a nitrocellulose membrane and analyzed by autoradiography.

*RRP43*, the yeast ortholog of the human *EXOSC8* has a low degree of conservation with the human gene, lacking the C-terminal region, where the human mutation p.Ser272Thr is located. Based on the observed mitochondrial dysfunction in patient V:10 we investigated whether a defect in the yeast ortholog of *EXOSC8* affects mitochondrial function. We took advantage of a promoter-shutoff *tetRRP43* strain^18^. Wild-type and *tetRRP43* cells were grown on non-fermentable carbon sources in the presence/absence of doxycycline 0.125 μg ml^−1^. Treatment with doxycycline caused a strong growth reduction on glucose of *tetRRP43* while only a slight reduction on glycerol suggesting that *RRP43* downregulation does not primarily affect oxidative phosphorylation capacity (Fig. 4a). We speculate that in the presence of glycerol, that slows cell growth and metabolic rate, the exosome complex is able to maintain a basal RNA abundance, despite the downregulation of *tetRRP43*. Furthermore, the reduced *RRP43* gene expression caused significantly decreased respiration that was paralleled by a decreased amount of mitochondrial cytochromes compared with wild type, although their structural integrity was not affected (Fig. 4c). No difference in mitochondrial translation rate was observed between wildtype and *tetRRP43* mutant in the presence/absence of doxycycline (Fig. 4d) suggesting that reduced expression of *RRP43/EXOSC8* may have a secondary effect on mitochondrial function, possibly due to disturbed mRNA processing of mitochondrial genes containing ARE elements^19^.

### Increased gene expression of selected ARE-containing mRNAs

**Figure 5:**
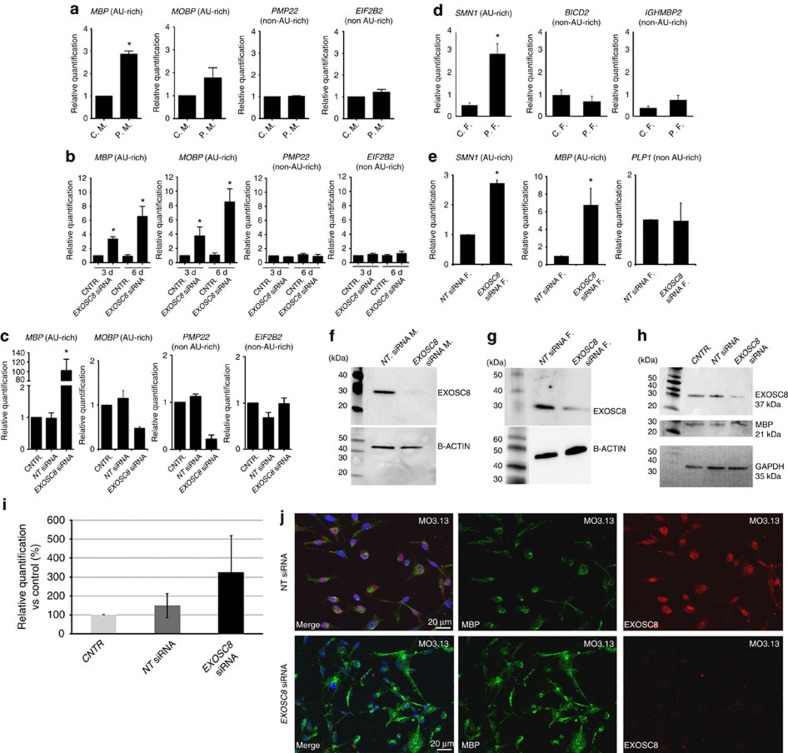
RT-PCR studies in patient myoblasts, control myoblasts and human oligodendroglia cells and immunohistochemistry and immunoblotting for myelin basic protein (MBP) in human oligodendroglia cells. (**a**) Quantitative PCR of *MBP, PMP22* and *EIF2B2* in patient and control myoblasts. The gene expression of *MBP* shows significant increase (fold change>2.99, *P*=0.0055) while no other gene expression is altered. C. M., control myoblasts; P. M., P1-V:10 myoblasts. (**b**) Relative quantification of *MBP* expression in human myoblasts treated with *EXOSC8* siRNA. Downregulation of *EXOSC8* resulted in increased expression of *MBP* and *MOBP*, but not in the non-AU-rich control genes *PMP22* and *EIF2B2*. C. M.,control myoblasts; CNTR., control; PP. M., P1-V:10 myoblasts;. (**c**) RT-PCR detected >100-fold increase of *MBP* mRNA in human oligodendroglia cells after *EXOSC8* downregulation, but no significant change was observed in mRNAs of the non AU-rich control genes *PMP22* and *EIF2B2*. Unpaired *t*-test was used for statistical analysis. Statistically significant changes are marked with *. Error bars show standard deviation of three experimental repetitions. (**d**) Quantitative PCR of *SMN1, BICD2* and *IGHMBP2* in patient and control fibroblasts. The gene expression of *SMN1* shows significant increase (~fourfold change, *P*=0.01984) while no other gene expression is altered. C. F., control fibroblasts; P. F., P3-II:1 fibroblasts. (**e**) Quantification of ARE-containing and non-ARE-containing genes in human fibroblasts after 3 days *EXOSC8* downregulation detected significant increase in *SMN1* and *MBP*. (**f**) Immunoblotting confirmed that *EXOSC8* siRNA downregulation resulted in severe depletion of this protein in both myoblasts (M) and (**g**) fibroblasts (F). (**h**) Immunoblotting (repeated three times) confirmed that siRNA downregulation of *EXOSC8* resulted in increased MBP protein levels in human oligodendroglia cells. CNTR., control; NT siRNA, non-targeting siRNA. (**i**) Quantification of MBP protein expression in differentiated oligodendroglia cells after *EXOSC8* downregulation. (**j**) Double immunolabelling of differentiated human oligodendroglia cells for MBP (green channel) and EXOSC8 (red channel). Left column: non-targeting control transfected cells. NT siRNA, non-targeting siRNA. EXOSC8 localized to the nucleus and MBP is distributed throughout the cytosol in EXOSC8 ablated cells. Right column: EXOSC8 staining confirms successful siRNA-mediated downregulation and increased MBP expression. Scale bar, 20 μm.

After the exclusion of a primary mitochondrial phenotype in RRP43 depleted yeast we studied the possible mRNA targets of *EXOSC8* in human cells. *EXOSC8* encodes a 3′–5′ exoribonuclease that specifically interacts with ARE-containing mRNAs suggesting that a defect in this protein might specifically alter degradation of ARE mRNAs. Based on the clinical presentation of predominant white matter abnormalities, cerebellar and corpus callosum hypoplasia, SMA and mitochondrial dysfunction in our patients we searched the ARE Database (ARED, http://brp.kfshrc.edu.sa/ARED/ ) with keywords ‘myelin’, ‘ataxia’, ‘spinal motor neuron’ and ‘mitochondrial’ (Supplementary Note 3). We studied mRNA levels of 18 selected ARE-containing and 16 non-ARE-containing genes in myoblasts and fibroblasts of our patients and in human control cells and oligodendroglia cells (MO3.13) before and after small-interferring RNA (siRNA) downregulation of *EXOSC8* (Fig. 5). EXOSC8-deficient patient myoblasts (P1-V:10) revealed a significant 2.99-fold increase (*P*=0.0055) in *MBP* gene expression compared with control human myoblasts (average of three different control cell lines), while the expression of the other selected genes did not show a significant change (Fig. 5a, Supplementary Table 2). *EXOSC8* downregulation in myoblasts significantly increased expression of two ARE-containing myelin-related genes: *MBP* (>6.5-fold, *P*=0.0167) (Fig. 5b, Supplementary Table 2) and myelin-associated oligodendrocyte basic protein (*MOBP* >8.5-fold, *P*=0.0158, Supplementary Table 2). However, no significant change was detected in mRNA levels of any of the other tested AU-rich and non-AU-rich genes (Supplementary Table 2). Based on the selective effect of *EXOSC8* downregulation on oligodendroglia-related genes, quantitative reverse transcription-PCR (qRT-PCR) analysis has been performed in *EXOSC8* downregulated human MO3.13 oligodendroglia cells (kind gift of Prof. Nalbantoglu, McGill). In support of our previous findings, we detected a highly significant increase in expression of *MBP* (>100-fold, *P*=0.013) (Fig. 5c), which led to increased MBP protein levels in differentiated oligodendroglia cells detected by immunostaining and immunoblotting (Fig. 5h–j).

In patient fibroblasts (P3-II:1) we detected a significant ~fourfold increase (*P*=0.01984) in the expression of *SMN1*, which is the major causative gene leading to SMA (Fig. 5d). No significant change was detected in the other studied genes (16 ARE and 16 non-ARE mRNAs), suggesting that only a subset of ARE transcripts was significantly affected by EXOSC8 dysfunction in both cell types. *EXOSC8* downregulation in fibroblasts resulted in significant increase of *SMN1* (2.72-fold, *P*=0.00038), *MBP* (6.74-fold, *P*=0.01936) and *MOBP* (2.48-fold, *P*=0.04767), but no significant change was detected in mRNA levels of any of the other tested AU-rich and non-AU-rich genes (Fig. 5e, Supplementary Table 3).

### Morpholino knock down of *exosc8* in zebrafish

**Figure 6:**
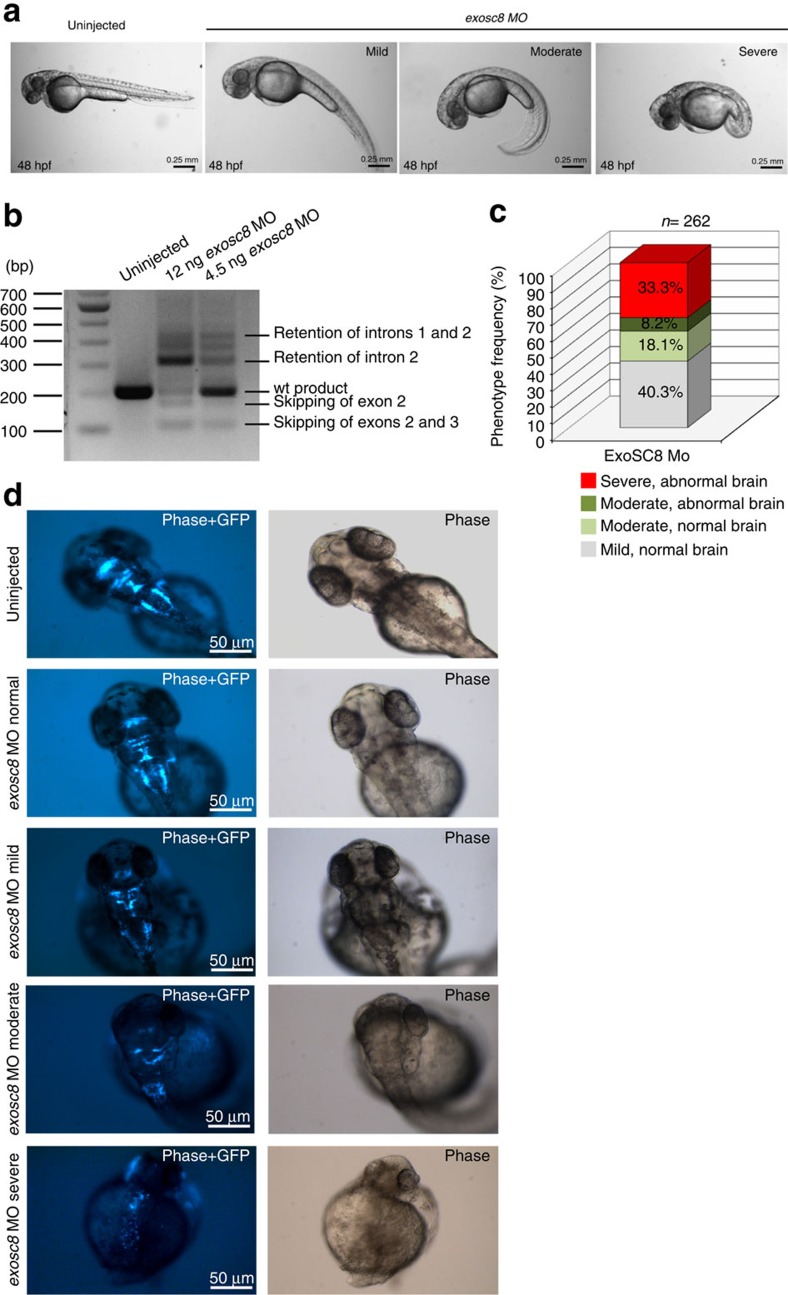
Knock down of the zebrafish *exosc8.* (**a**) Morphology of live embryos at 48 hpf injected with 12 ng splice-blocking *exosc8* antisense MO at the 1–2 cell stage. From left to right: un-injected control embryo, *exosc8* MO injected mild, moderate and severe phenotype, respectively. Mild phenotype: slightly curved tail; moderate phenotype: C-shaped; severe category: abnormally formed, very short tail, cardiac oedema, and small, misshapen or missing eyes. (**b**) RT–PCR analysis: analysis of *exosc8* transcripts from embryos injected with the splice-blocking *exosc8* MO which targets the splice donor site of exon 2. Using primers in exons 1 and 4, RT–PCR yielded several additional bands in MO injected embryos originating from mis-spliced transcripts. Wild-type transcript is still present in embryos injected with 4.5 ng of MO, but only a trace of wild-type product is left in embryos injected with 12 ng of MO, therefore 12 ng of MO was used in subsequent experiments. wt, wildtype. (**c**) Relative distribution of the *exosc8* morphant phenotypes described above. External morphology described in **a** and cranial nerve abnormalities displayed in panel **d** are both taken into account in this categorization; *exosc8* MO was injected in three independent experiments into embryos of the Tg(islet-1:GFP) strain and a total of 262 MO injected embryos were evaluated by light and fluorescent microscopy. (**d**) Brain abnormalities of *exosc8* morphants (Tg(islet-1:GFP) strain) from the different phenotypes at 48 hpf: dorsal views of GFP positive neurons in the midbrain and the hindbrain. These transgenic zebrafish embryos express GFP in cranial motor and sensory neurons and in the efferent neurons for the lateral line and the vestibule-acoustic nerves. Normal cranial neuron structure and development was detected in embryos of the normal and mild category. Disrupted neuronal structure in the severe and in a proportion of the moderate categories; GFP positive cells are scattered, no clear structures are visible and overall GFP expression is reduced.

To study the link between reduced EXOSC8 and the human disease we established a zebrafish model for EXOSC8 deficiency. Zebrafish has previously been used to model human demyelination and pontocerebellar dysfunction^20^,^21^,^22^. We selected two different antisense morpholino oligonucleotides (MO) to downregulate the zebrafish *exosc8* gene (Gene ID:323016, mRNA:NM_001002865, Supplementary information) in embryos from the golden strain and from the islet-1-green fluroscent protein (GFP) transgenic zebrafish line (Tg(islet-1:GFP)^23^. Confirming the efficiency of the splice-blocking *exosc8* MO, RT-PCR indicated the loss of wild-type *exosc8* transcript (Fig. 6b, Supplementary Fig. 2). In support of the phenotype caused by the splice-blocking *exosc8* MO, we detected similar changes by using the second translation-blocking *exosc8* MO in zebrafish. Knock down of *exosc8* in zebrafish embryos resulted in a set of phenotypes ranging from mildly to severely abnormal external morphology (Fig. 6a,c). Swimming abilities and touch-evoked escape response at 48 h post fertilization (hpf) were impaired even in embryos with a mild phenotype (Supplementary videos). Importantly, downregulation of *exosc8* led to abnormal brain development in islet-1-GFP transgenic zebrafish, which express GFP in the motor neurons of the hindbrain. The degree of brain abnormality correlated with the increased severity of morphant phenotype (Fig. 6d), suggesting that Exosc8 is essential for brain development in zebrafish. Although the recapitulation of an identical phenotype with translation-blocking MO is in support of a specific effect in our experiments, to exclude an off-target effect we introduced co-injection of *p53* MO to block off-target cell death.

We performed several attempts to rescue the phenotype of MO-treated zebrafish with different doses of wild-type human *EXOSC8* mRNA. We observed a dose-dependent toxic reaction after addition of the wild-type *EXOSC8* mRNA to MO-treated zebrafish. The reason behind the toxicity may be that an imbalanced supply of the different exosomal proteins may have deleterious effect on the function of the exosome, as comparative assessment of protein abundance and localization in living cells showed, that EXOSC8 and EXOSC9 are present in a 1:1 stoichiometry within the complex^24^.

**Figure 7:**
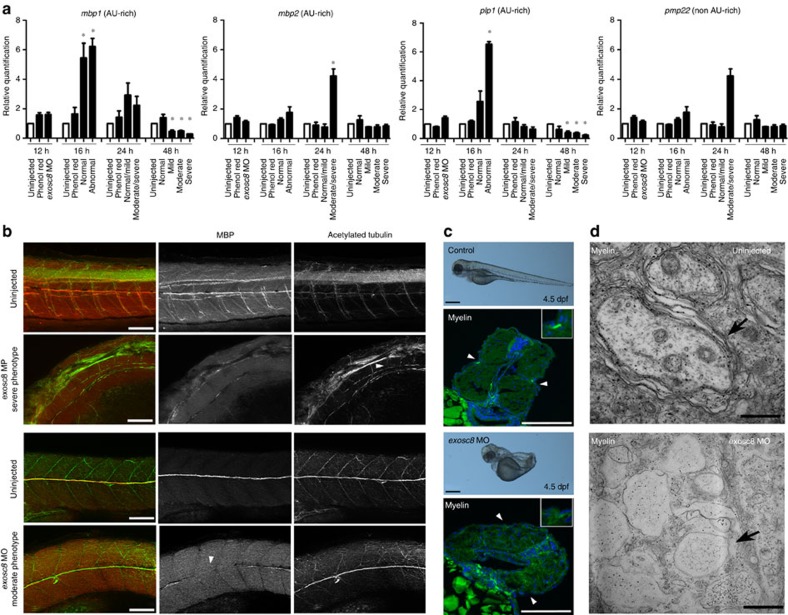
MBP and acetylated tubulin staining after knock down of the zebrafish ortholog: *exosc8.* (**a**) *exosc8* MO injected larvae were analyzed for expression of AU-rich mRNAs during 48 hpf by real-time PCR. At 16 hpf expression of *mbp1* and *plp1* was increased in embryos with an abnormal phenotype, with a similar increase in *mbp2* observed at 24 hpf. Despite this initial increase, by 48 hpf there is a dramatic decrease in *mbp1* and *plp1* expression in larvae with a moderate and severe phenotype. Each bar or severity group at different timepoints represents a number of 15–20 embryos. Statistically significant changes (*P*<0.05) are marked with *. Unpaired *t*-test was used for statistical analysis. Error bars represent s.d. of three experimental repeats. (**b**) Un-injected control larvae and *exosc8* MO injected larvae were analyzed for myelination at 96 hpf. Larvae were stained with antibodies against the zebrafish MBP and against acetylated tubulin. Left column: overlay, MBP staining in red, acetylated tubulin staining in green; middle column: MBP staining; right column: acetylated tubulin. Top row: tail of control larva: motor axons in each somite are clearly visible and myelinated at 96 hpf. Second row: tail of MO injected larva with a moderate phenotype: the spinal cord is curved and has an irregular structure. Motor axons in the somites are either very short (arrowhead) and thin or missing completely and are not MBP-positive. Third row: un-injected control larva, posterior lateral line, intact myelin. Bottom row: *exosc8* MO injected larva with moderate phenotype: the lateral line is present (green acetylated tubulin signal) but the myelination of its neurons is interrupted (arrowhead). Scale bar, 100 μm. (**c**) Myelin staining of the lateral line was studied in control un-injected and *exosc8* MO injected zebrafish larvae at 4.5 dpf. Representative images of the analyzed embryos are shown on the top (scale bar, 0.25 mm) and transverse sections of the embryos are shown on the bottom. In the control larvae the myelinated lateral line is present at both sides (white arrowheads). However, no myelination of the lateral line was detected in the *exosc8* MO injected larvae (white arrowheads). Higher magnifications are shown in the upper right hand corners. Scale bar, 100 μm. (**d**) Representative EM pictures of the myelin sheath at the lateral line in un-injected and *exosc8* MO injected zebrafish larvae at 4 dpf. Black arrows indicate the myelin sheet around the axon. Scale bar, 500 μm.

Similar to the studies performed in human cells, we analyzed AU-rich mRNAs encoding myelin proteins in *exosc8* morphants and control zebrafish embryos. Similar to the results in human oligodendrocytes and myoblasts, *mbp* mRNA increased initially (16 hpf) in *exosc8* morphants, but decreased at later timepoints (48 hpf), especially in morphants with severe phenotype, most likely due to the loss of neuronal structures and surrounding oligodendroglia (Fig. 7a). Previous studies^25^ and the ZFIN database detected expression of *mbp* by *in situ* hybridization during early somitogenesis at about 11 hpf (http://zfin.org/cgi-bin/webdriver?MIval=aa-fxfigureview.apg&OID=ZDB-FIG-050630-1515), and *exosc8* expression at the 14–19 somite stage around 16 hpf (http://zfin.org/cgi-bin/webdriver?MIval=aa-fxallfigures.apg&OID=ZDB-PUB-040907-1&fxallfig_probe_zdb_id=ZDB-EST-080225-97). We did not study expression of the zebrafish *smn1* gene, because in contrast to the human *SMN1* zebrafish *smn1* is not ARE.

We also performed whole-mount immunohistochemistry in zebrafish larvae after *exosc8* MO injection with an antibody against the zebrafish MBP (kind gift of Prof. Talbot, Stanford). In un-injected embryos, MBP expression at 4 days post fertilization (dpf) was observed in the ventral hindbrain, spinal cord, along motor axons exiting the spinal cord and the lateral line. In *exosc8* morphants the MBP signal on motor axons in somites, on the posterior lateral line and in the head was missing or interrupted (Fig. 7b, Supplementary Fig. 3). We also performed combinatorial labelling (BrainStain Imaging Kit, Molecular Probes) including FluoroMyelin Green stain, which works via the high lipid content of myelin and provided further evidence, independent from immunostaining with MBP, that myelination is primarily impaired in *exosc8* MO downregulated zebrafish (Fig. 7c). As a final proof of a defective myelination, electron microscopy (EM) on *exosc8* MO-treated zebrafish revealed a clear defect in myelination at 4 dpf (Fig. 7d).

**Figure 8:**
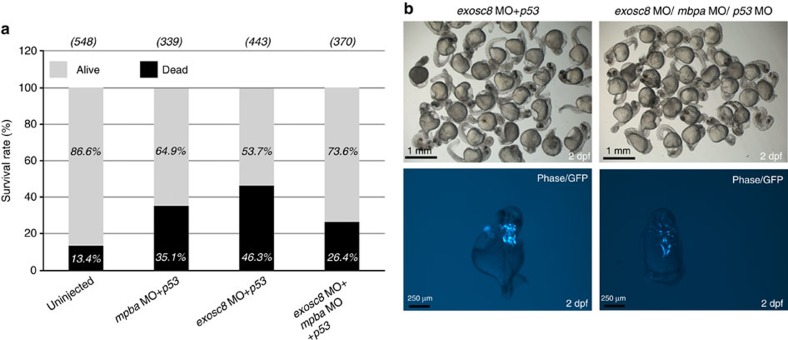
Simultaneous knock down of *exosc8/mbpa/p53* in zebrafish. (**a**) Representative graph shows the survival rate of un-injected, *mpba+p53*, *exosc8+p53* and combined *mbpa+exosc8+p53* MO injections of the Tg(islet-1:GFP) strain at 48 hpf (summary of 3 experiments). The survival rate increased after triple MO injections compared with *exosc8+p53* knockdown embryos. (**b**) Top row: morphology of the severe embryos at 48 hpf injected with *exosc8+p53* and *exosc8+mbpa+p53*. Bottom row: brain abnormalities of *exosc8+p53* and *exosc8+mbpa+p53* morphants (Tg(islet-1:GFP) strain) at 48 hpf. Dorsal views of GFP positive neurons in the midbrain and the hindbrain indicate abnormal brain structures in severe *exosc8+p53* morphants. Improved cranial neuron structure was detected in severe *mbpa+exosc8+p53* MO injected embryos. The same result has been reproduced in three separate experiments with equal MO doses, on the same clutch of embryos.

Simultaneous MO knock down of zebrafish *mbpa* (gene ID: 326281, NC_007130.5) and *exosc8* was performed to explore whether the phenotype of *exosc8* downregulated zebrafish embryos can be rescued by knocking down *mbp*, which is originally increased in *exosc8* downregulated zebrafish. Survival rate of MO injected zebrafish embryos was better in the simultaneous *mbpa, exosc8* knockdown group (73.6%) compared with embryos treated with *exosc8* MO only (53.7%) (Fig. 8a). Furthermore, in embryos with severe phenotype the pattern of midbrain and hindbrain nuclei was slightly more preserved in simultaneous *mbpa, exosc8* MO-treated embryos compared with zebrafish treated with *exosc8* MO only (Fig. 8b). Taken together, these data suggest that the neuronal defect caused by *exosc8* downregulation primarily impairs myelination by affecting the regulation of *mbp* genes. Furthermore, we show that additional knock down of *mbp* improved the survival rate and brain structure of *exosc8* MO downregulated zebrafish, suggesting that the original increase of *mbp* expression may trigger downstream events resulting in loss of MBP and impaired myelination in zebrafish.

## Discussion

The first human disease linked to an integral exosome component was EXOSC3 deficiency, which has been identified in pontocerebellar hypoplasia (PCH) and spinal motor neuron disease (PCH1, MIM 607596)^26^,^27^,^28^. A broader clinical spectrum was recently suggested in patients with *EXOSC3* mutations including isolated cerebellar hypoplasia and spinal anterior horn involvement or intellectual disability, early-onset spasticity and cerebellar atrophy^29^. A severe form of PCH1 has been reported among Czech Roma due to a founder mutation in *EXOSC3* (ref. 30).

We discovered pathogenic mutations in the exosomal protein gene *EXOSC8* in 22 patients with profound infantile neurodegenerative disease combining features of cerebellar and corpus callosum hypoplasia, hypomyelination and SMA. The similar disease spectrum caused by the defect of a different exosome protein suggest that impaired mRNA metabolism due to exosome dysfunction is the major common pathomechanism. However, while *EXOSC3* mutations affect mostly spinal motor neurons and Purkinje cells, oligodendroglia cells are also targets of mutations in *EXOSC8.* It is possible that different exosome components affecting different ARE mRNAs, potentially based on their length^9^, or mRNA metabolism may have neuronal cell-type specific differences, potentially influenced by the type or localization of the exosome defect. However, both studies clearly suggest the major role of ARE mRNA metabolism in this complex neurodegenerative disease.

In support of a close interaction between EXOSC8 and EXOSC3 within the exosome, reduced EXOSC3 protein levels were detected in our patients carrying *EXOSC8* mutations, and also in control cells after siRNA downregulation of *EXOSC8* (Supplementary Fig. 4).

Both peripheral and central myelination requires an exact ratio of several myelin proteins^31^. Central nervous system myelin is a multi-layered membrane sheath generated by oligodendrocytes for rapid impulse propagation. It has been recently shown that new myelin membranes are incorporated adjacent to the axon, and simultaneously, newly formed layers extend laterally, leading to the formation of a set of closely apposed paranodal loops^32^. This model can explain the assembly of myelin as a multi-layered structure, where increased amount of MBP, a peripheral membrane protein, may result in premature compaction and may block myelin growth^32^ or compaction at aberrant locations, which could also be toxic for myelination^22^. Duplication of *PLP1* is a frequent cause of PMD^33^,^34^,^35^ and a similar gene dosage effect of *PMP22* is responsible for the demyelinating peripheral neuropathy in Charcot-Marie-Tooth disease type 1A^36^. A link between ARE mRNA decay and demyelination is further supported by the progressive leukodystrophy caused by mutations in the AU-specific RNA binding protein (AUH)^37^.

We suggest that increased mRNA levels of ARE-containing myelin proteins (MBP, MOBP) resulting from *EXOSC8* deficiency initiate a cascade of downstream events, and the disturbed balance between ARE and non-ARE myelin components results in demyelinating disease. Ultimately, the loss of oligodendroglia cells leads to a secondary decrease of myelin proteins, as shown in exosc8 MO injected zebrafish, where an initial increase, followed by a secondary decrease of myelin proteins resulted in defective myelination.

The genetic causes of isolated PCH are autosomal recessive mutations in genes involved in transfer RNA splicing and processing^38^. Splicing of the pre-mRNAs by the spliceosome depend on small nuclear ribonucleoproteins, which require Spinal Motor Neuron 1 (SMN1) protein for their assembly and defect of SMN1 results in SMA, a leading cause of infantile mortality^39^. The exact mechanisms behind the strict cell-type specific effect of these factors involved in RNA metabolism are still unclear; however, it is possible that some clinical presentations of *EXOSC8* and *EXOSC3* mutations, and potentially other human exosome related diseases, are due to abnormal RNA splicing.

In summary, patients with *EXOSC8* mutations present with a characteristic spectrum of overlapping phenotypes of infantile onset hypomyelination, cerebellar hypoplasia and SMA. The clinical presentation may depend on the type and localization of mutations and provides a clue to unravel the complex molecular pathways caused by the defective exosome function.

## Methods

### Patients

We have received informed consent from the patients (parents) included in this study. Ethical approval was obtained from the County Durham and Tees Valley 2 Research Ethics Committee (08/H0905/106) and from the Ethics committee of the Hebrew University Hadassah Medical School (0485-09). Written consent to publish the photo of a patient was obtained.

### Histological and biochemical analyses of skeletal muscle

Histological and biochemical analyses of skeletal muscle were performed by standard methods^40^.

### Autopsy staining methods

The brain and spinal cord samples were fixed in 10% phosphate-buffered formal saline before embedding in paraffin wax. For Luxol Fast Blue staining 10-μm sections were dehydrated and stained with Luxol Fast Blue overnight at room temperature, and then rinsed in alcohol and water to remove excess blue colour. The sections were differentiated with a weak solution of lithium carbonate, followed by 70% alcohol solution. Immunohistochemistry was done following citrate (pH 6) and EDTA (pH 8) pre-treatment for 10 min. Immunohistochemistry was carried out on 5-μm sections with mouse monoclonal antibodies that recognize MBP (1:2000 SM194R, Covance, NJ, USA) and vimentin (1:5600 clone V9 Dako; Copenhagen, Denmark). Rabbit monoclonal antibody was used for p62 1:150 SQTM1 (Santa Cruz Biotechnology, TX, USA). Sections were also stained with haematoxylin. Stainings were performed on at least three different sections.

### Genetic analysis

Genome-Wide Human SNP Array 6.0 (Affymetrix) was performed in pedigree 1 on six affected (P1-V:2, P1-V:10, P1-V:19, P1-V:22, P1-V:26, P1-V:28) and one unaffected family member (P1-IV:14), and in pedigree 3 and in 2 it was performed on affected family members (P3-II:1, P3-II:3) Single nucleotide polymorphisms were analyzed using HomozygosityMapper^13^. Excess homozygosity was defined using [max_block_length=1,000] and an excess homozygosity threshold of 0.8 (80%)^13^. For exome sequencing genomic DNA from patients P1-V:10, P1-V:29 from pedigree 1 and P3-II:1 from pedigree 3 was fragmented to 150–200 bp with the use of Adaptive Focused Acoustics (Covaris); then end-repaired, adenylated, and ligated to adaptors (Illumina Paired-End Sample Preparation kit). Ligated libraries were hybridized with whole-exome baits that covered 27,184 genes with modifications for the SureSelect Human All Exon Kit Version 2 (Agilent), Illumina Paired-end Sequencing Library Version 2.0.1. The captured fragments were purified and clonally amplified, and then sequenced on two lanes of an Illumina Genome Analyzer IIx with the use of 75 bp paired-end reads. Sequence was aligned to the human reference genome (UCSC hg19) with the Burrows Wheeler Aligner^41^,^42^, then reformatted with the use of SAMtools v0.1.18 (ref. 43). Exon target sequence (93.4%) was covered by >10 reads with a mean target depth of 121. Single base variants were identified with Varscan v2.2 (ref. 44) and Indels were identified with Dindel v1.01 (ref. 45). Variants were annotated using wAnnovar^46^. Lists of on-target variants were filtered against data from NHLBI-6500-ESP, the 1,000 Genomes project, and the exome sequences of 334 unrelated in-house control exomes to identify rare homozygous variants with a MAF<0.01.

### Cell culture and siRNA transfection

Cultured myoblasts of patient V:10 (pedigree 1) and controls were grown in skeletal muscle cell growth medium and supplement mix (PromoCell) supplemented with 10% (v/v) foetal bovine serum (FBS, Sigma Aldrich) and 4-mM L-glutamine (Invitrogen) and cultured as recommended by the supplier. Fibroblasts of patient P3-II:1 were grown in high glucose Dulbecco’s modified Eagle’s medium (DMEM, Sigma, Poole, UK) supplemented with 10% FBS. Myoblasts of patient V:10 and a control cell line were immortalized by transduction with a retroviral vector expressing the catalytic component of human telomerase^47^. *Silencer* *EXOSC8* RNA (s22370 siRNA, Ambion–Life Technologies) was transiently transfected in control myoblasts, fibroblasts and in M03.13 human oligodendroglia cells at a final concentration of 5 nm using Lipofectamine RNAiMAX (Invitrogen), according to the manufacturer’s specifications. Transfections were repeated on day 3 and cells were harvested on day 6. A non-targeting *Silencer*Select Negative Control (#1) was used as a control.

Human oligodendrocytic cells (MO3.13) were grown in DMEM supplemented with 10% FBS. The human oligodendroglia cell line was received from the McGill University, where it was originally characterized. Medium was changed every 2–3 days. MO3.13 cells were differentiated in oligodendrocyte differentiation medium for 7 days. Each experiment was repeated at least three times.

### Immunoblotting

Aliquots of total protein were loaded on 14% SDS–polyacrylamide gel electrophoresis (SDS–PAGE), transferred to polyvinylidene fluoride membranes and subsequently probed with a polyclonal antibody recognizing EXOSC8 (Proteintech Group Inc., 11979-1-AP, 1:200) or Pierce Antibodies (PA5-12378, 1:100, Fig. 3f) and monoclonal antibodies targeting mitochondrial COXI (Mitosciences MS404, 0.5 μg ml^−1^), COXII (Abcam ab110258, 1 μg ml^−1^), NDUFB8 (Abcam ab110242, 0.5 μg ml^−1^), MBP (Covance SMI-94R, 1:1,000), EXOSC3 (Proteintech Group Inc., 15062-1-AP, 1:200), GAPDH (Santa Cruz sc25778, 1:500), porin (Abcam ab14734, 1 μg ml^−1^) and β-actin (Sigma A1978, 0.5 μg ml^−1^), anti-acetylated tubulin for zebrafish (Sigma, T 6793, 1:500) according to the instructions of the suppliers. MBP for Zebrafish was a kind gift from Prof. William Talbot. Each experiment has been repeated at least three times.

### Blue native PAGE (BN–PAGE)

BN–PAGE was performed as described^48^.

### Yeast strains and culture condition

*Saccharomyces cerevisiae* strains used in this study are *R1158* (deletion consortium wild-type *MATa his3Δ1 leu2Δ0 met15Δ0 ura3Δ0 BY4741* with TTA integrated at URA3) and *RRP43* Tet-O_7_-promoter mutant (referred as *tetRRP43*), kindly provided by T. Hughes^18^. The promoter-replacement system approach allows modulating the downregulation of essential genes by replacing the native promoter with one tetracycline-regulatable promoter that can be repressed by addition of doxycycline to the growth medium.

Cells were cultured in yeast peptone (YP) medium (1% Bacto–yeast extract and 2% Bacto–peptone (ForMediumTM, UK))^49^. Various carbon sources were added at 2% (w/v) (Carlo Erba Reagents, Italy). Media were solidified with 20 g l^−1^ agar (ForMediumTM, UK). For respiration and mitochondria extraction, cells were grown to late-log phase in the YP medium supplemented with 0.6% glucose. Oxidative growth, respiration and cytochrome spectra in yeast were performed. Oxidative growth phenotype was performed by spotting decreasing concentrations of yeast cells on YP medium supplemented either with 2% glucose or with 2% glycerol. Differential spectra between reduced and oxidized cells of a suspension of cells at 500 mg ml^−1^ (wet weight) were recorded at room temperature, using a Cary 300 Scan spectrophotometer (Varian, Palo Alto, CA, USA). Oxygen uptake rate was measured at 30 °C using a Clark electrode in a reaction vessel of 3 ml of air-saturated respiration buffer (0.1 M phthalate–NaOH, pH 5.0), 10 mM glucose, starting the reaction with the addition of 10 mg of wet weight of cells^50^. Mitochondrial protein synthesis in yeast was performed by standard methods^51^.

### Gene expression studies

cDNA was generated by reverse transcription of 500 ng of total RNA using the Superscript VILO cDNA synthesis kit (Invitrogen, 11754-050) according to manufacturers’ instructions. qPCR (Applied Biosystems 7900HT) was performed in triplicate on cDNA using SYBR Green PCR Master Mix (Invitrogen, 4309155). For human cells samples were normalized using the average of three reference genes, GAPDH, β–tubulin and actin; and for zebrafish samples were normalized to actin, EF1α and Rpl13α. Primers are shown on Supplementary Table 4.

### Measurement of *EXOSC8* mRNA copy numbers

Patient and three control fibroblast lines were grown in DMEM supplemented with 15% FBS and 1% Penicillin/Streptomycin to semi-confluence. To half of the cultures 300 mM puromycin was added 6 h prior to harvesting the cells. mRNA was prepared with the Trizole method and reverse transcribed into cDNA with random hexamers using the Superscript II kit (Life Technologies). For qPCR detection we used SYBR Green (Life Technologies) and a primer pair that generated a 198-bp product that comprised all known splice isoforms of *EXOSC8*: forward, 5′-GCATCCGTGTCGAAAGTGC-3′; reverse, 5′-CTGGGTTCAAAACCGTGGAACC-3′. The qPCR was run on an ABI 7700 machine using the –ΔΔC_t_ method with *HPRT*: forward, 5′-ACAATGCAGACTTTGCTTTCC-3′; reverse, 5′-TCAAGGGCATATCCTACAACAA-3′) as the reference gene.

### Zebrafish strains and maintenance

Zebrafish strains used in this study were the golden strain and the islet-1-GFP transgenic line (Tg(islet-1:GFP), expressing GFP driven by the islet-1 promoter)^23^. All zebrafish procedures were performed under Home Office UK licence regulations. Zebrafish embryos were collected and raised at 28.5 °C according to standard procedures^52^ and staged in hpf or dpf according to standard criteria^53^ PTU (0.003%; Sigma) was used to suppress pigmentation when necessary. The *p53* morpholino was purchased from Gene Tools (Pilomath, OR).

### Antisense MO injections

The antisense MO to downregulate *exosc8, mbpa* and *mbpb* expression in zebrafish was purchased from Gene Tools. The MO was designed using the mRNA of the zebrafish exosc8 orthologue; (Gene ID: 323016, mRNA accession number: NM_001002865) and the corresponding genomic sequence on zebrafish chromosome 10. We designed a splice-blocking MO directed against the splice donor site of exon 2: 5′-AGATTAACTCTCACCAGAAAGCTCC-3′ and a second translation-blocking MO 5′-TTTAAAACCAGCCGCCATGATGTTT-3′. Embryos were injected with 10–12 ng *exosc8* MO. We designed a translation-blocking MO directed against *mbpa* (5′-GGCCATTCTAGGTGTTGATCTGTTC-3′). Embryos were simultaneously injected with 1 ng MO, with the addition of 5-ng *p53* MO. We also simultaneously injected *mbpa* (1 ng) and *exosc8* (10–12 ng) MOs with 5 ng *p53* in separate experiments with equal MO doses, on the same clutch of embryos.

Three independent MO injection experiments were performed and over 300 injected embryos were evaluated in total to establish morphant phenotypes. MO injection into embryos of the Tg(islet-1:GFP) strain were used to study brain abnormalities, whereas embryos of the *golden* strain were used for whole-mount antibody staining procedures. Zebrafish were anesthetized with Tricaine solution and phenotyped at 48 hpf to assess brain and tail morphology. Images were captured using a MZ16F fluorescent stereomicroscope (Leica).

### RNA isolation and RT-PCR in zebrafish

RNA from zebrafish embryos and larvae was isolated with Trizol reagent (Invitrogen) following the manufacturer’s instructions. For RT–PCR analysis in developing zebrafish embryos and following MO injection, RNA from ~30 embryos was extracted and after cDNA synthesis from three different MO injections. Primers used for zebrafish RT–PCR are shown on Supplementary Table 5.

### Whole-mount antibody immunofluorescence

For whole-mount immunofluorescence staining, zebrafish embryos and larvae were fixed in 4% paraformaldehyde in phosphate-buffered saline overnight at 4 °C and then permeabilized in cold acetone at −20 °C. In addition, 4-dpf-old larvae were permeabilized with collagenase A (Roche Diagnostics, 1 mg ml^−1^) for 90 min. Embryos/larvae were blocked in 5% horse serum in phosphate-buffered saline containing 0.1% Tween-20 (PBT). Embryos/larvae were incubated in blocking solution containing primary antibody overnight at 4 °C followed by washing several times with PBT and incubation with secondary antibody (anti-rabbit Alexa Fluor 594 and anti-mouse Alexa Fluor 488, Invitrogen). The primary antibodies used in this study were a polyclonal rabbit anti-MBP antibody (1:50 dilution, a kind gift from Prof. William Talbot, Stanford University) and a mouse monoclonal anti-acetylated tubulin antibody (1:500 dilution, Sigma) to label axon tracts. Combinational staining of myelin, neurons and nuclei in zebrafish embryos was performed using BrainStain Imaging Kit (Molecular Probes, B34650). Stained embryos/larvae were imaged with a Zeiss Axio Imager microscope. Immunofluorescence images were collected using a Zeiss Axio Imager Z1 fluorescence microscope equipped with Zeiss Apotome 2 (Zeiss, Germany) in AxioVision Rel 4.9 software.

### Electron microscopy

Zebrafish at 4 dpf were fixed in 2% glutaraldehyde in sodium cacodylate buffer at 4 °C overnight and suddenly washed three times (15 min each) in cacodylate buffer, and then stained with 1% osmium tetroxide (Agar Scientific) in dH2O for 1 h. Fish were dehydrated using graded acetone (25, 50 and 75% and twice in 100%). Fish were impregnated through increasing concentration of resin in acetone (25, 50, 75 and 100%) and then embedded in 100% resin at 60 °C for 24 h (TAAB Lab. Equip). Ultra-thin transverse sections of ~70 nm were cut using a diamond knife on a Reichert Ultracut E ultramicrotome. The sections were stretched with chloroform to eliminate compression and mounted on pioloform-filmed copper grids. The grids were then stained with 2% aqueous uranyl acetate lead citrate and subsequently examined using a Philips CM 100 Compustage (FEI) Transmission Electron Microscope and digital images were collected using an AMT CCD camera (Deben) at the Electron Microscopy Research Services, Newcastle University.

### Statistical analysis

For the statistical analysis of qRT-PCR we used unpaired *t*-test.

## Author contributions

V.B., J.S.M., A.Py. and J.M. were involved in the data acquisition, study design and the drafting of the manuscript. V.B. performed studies in oligodendrocytes and imaging in zebrafish. J.S.M. was responsible for the majority of the zebrafish work. A.Py. performed and analyzed the genetic studies (homozygosity mapping, exome sequencing, Sanger sequencing). J.M. did the real-time PCR work to study ARE mRNAs in humans and zebrafish. J.Q. and I.F. designed and performed the experiments in yeast. T.D., V.K., A.Pr., H.P. and S.E. provided the pedigrees and patient information. M.G. performed a part of the zebrafish work. T.P. has done the autopsy analysis. D.B. reported the MRIs. M.R. performed the immortalization of patient cells. M.S., S.L. and Y.C. performed cDNA studies and immunoblotting of patient fibroblasts. L.F. and L.K. provided the Roma haplotype data. M.S.K. and H.G. did the bioinformatics analyses. H.L., P.F.C, D.J.E., O.E. and S.E. were involved in the study design and in drafting the manuscript. R.H. initiated and organized the study and drafted and finalized the manuscript.

## Additional information

**How to cite this article:** Boczonadi, V. *et al*. EXOSC8 mutations alter mRNA metabolism and cause hypomyelination with spinal muscular atrophy and cerebellar hypoplasia. *Nat. Commun.* 5:4287 doi: 10.1038/ncomms5287 (2014).

**Accession codes:** Patient exome sequence data have been deposited in the European Genome-phenome Archive (EGA) under the accession code EGAS00001000856.

## Supplementary Material

Supplementary Figures, Tables and Notes Supplementary Figures 1-5, Supplementary Tables 1-5 and Supplementary Notes 1-3

Supplementary Movie 1 Motility of un-injected control zebrafish embryos at 48 hpf. This video shows normal motility.

Supplementary Movie 2 Motility of exosc8 MO treated embryos is impaired at 48 hpf. Morphants from the mild or moderate groups start circling when touched or only twitch; morphants from the severe category are paralyzed.
