# Supplementary material for: EXOSC8 mutations alter mRNA metabolism and cause hypomyelination with spinal muscular atrophy and cerebellar hypoplasia
Source: Nat Commun. 2014 Jul 3;5:4287. doi: 10.1038/ncomms5287 (PMC4102769; doi:10.1038/ncomms5287)
Supplement: Supplementary Figures, Tables and Notes — Supplementary Figures 1-5, Supplementary Tables 1-5 and Supplementary Notes 1-3 [file ncomms5287-s1.pdf]

Supplementary Information

Supplementary Figure 1

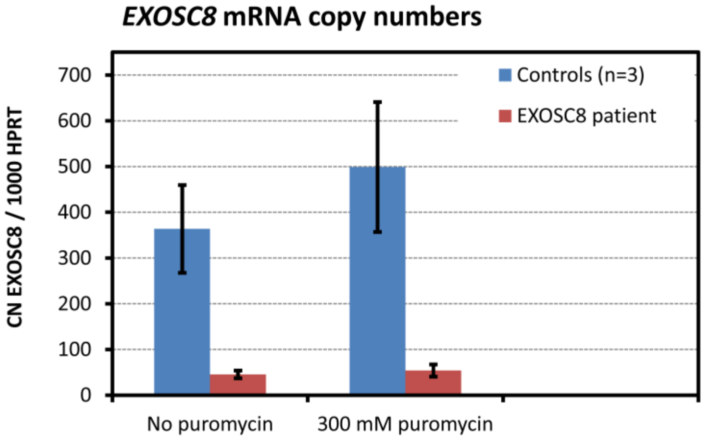

RT-qPCR analysis of *EXOSC8* mRNA copy numbers in fibroblasts of a patient and controls.

The c.5C>T mutation destabilizes the *EXOSC8*-mRNA, which is reduced to less than 10% of the wild=type levels. The mRNA decay is not caused by classic nonsense mediated messenger decay, because there is no increase in mutant *EXOSC8* mRNA copy number after 300 mM puromycin treatment. The mRNA instability might be caused by a problem of the 5'-capping / de-capping of the mRNA. The binding of the de-capping enzyme and its access to the cap structure competes with the binding of the translation initiation complex. If the latter does not bind properly, the mRNA might be degraded before it is translated. Columns depict the mean ± SEM (n=3 controls and n=1 patient, each measured in 3 independent experiments).

Supplementary Figure 2

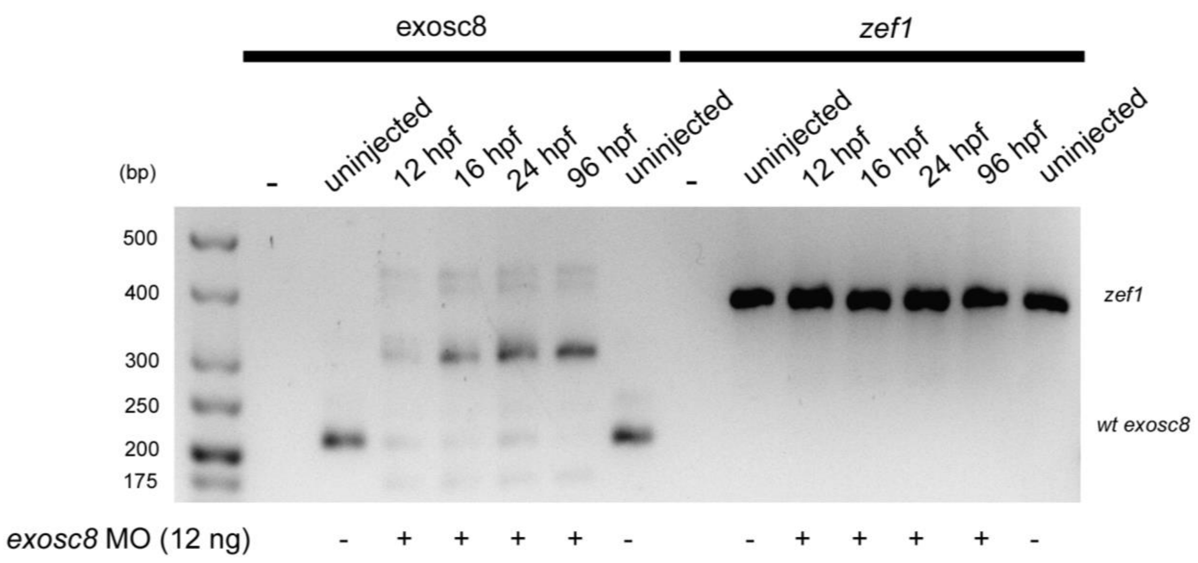

RT-PCR analysis of *exosc8* and *mbp* transcripts at various developmental stage.

Analysis of *exosc8* transcripts from embryos injected with the splice blocking *exosc8* MO which targets the splice donor site of exon 2. Using primers in exons 1 and 4, RT-PCR yielded several additional bands in MO injected embryos originating from mis-spliced transcripts at 12, 16, 24 and 96 hpf. Wild type transcript is present in un-injected embryos, but only a trace of wild type product is left in embryos injected with 12 ng of MO. Transcript of *zef1* was used as a housekeeping gene.

Supplementary Figure 3

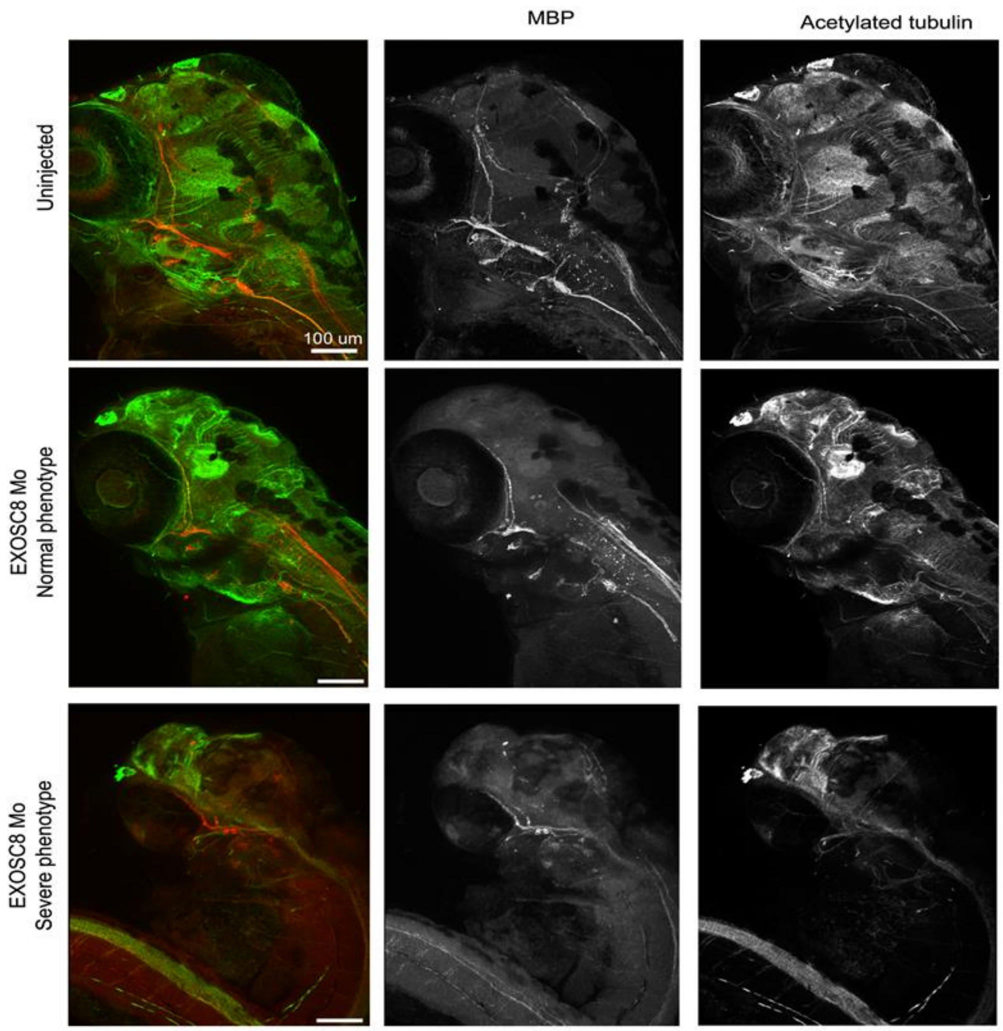

Mbp and acetylated tubulin staining after knock-down of the zebrafish orthologue, *exosc8* in the head

Un-injected control larvae and *exosc8* MO injected larvae were analysed for myelination at 96 hpf. Larvae of the different phenotype categories and control larvae were stained with antibodies against the myelin basic protein (Mbp) and against acetylated tubulin to study the presence of axon tracts and their degree of myelination.

Left column: overlay, with Mbp staining in red and acetylated tubulin staining in green; middle column: Mbp staining; right column: acetylated tubulin. Top row: head of control larva. Middle row: MO injected larva with normal external morphology; axons near the eye are not Mbp-positive (white arrowhead). Bottom row: MO injected larva with a severe phenotype.

Supplementary Figure 4

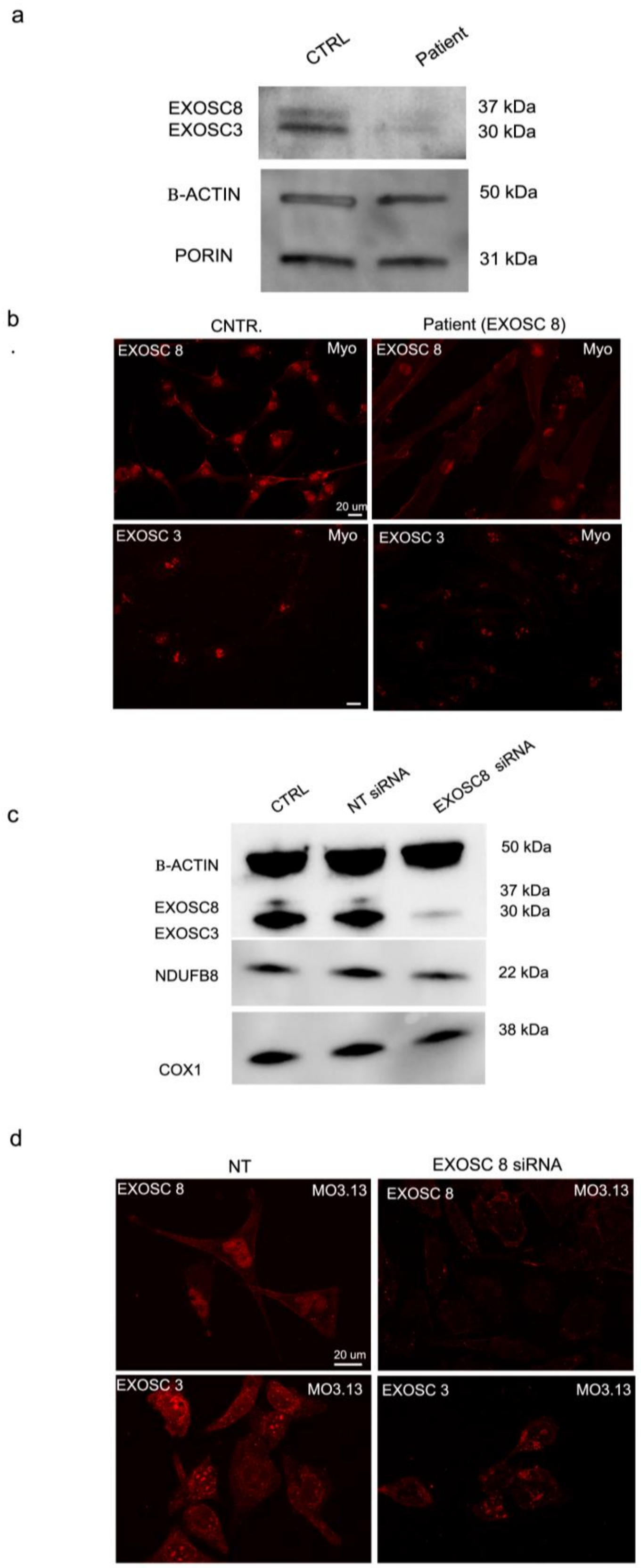

Immunoblotting and immunohistochemistry for EXOSC8 and EXOSC3 in human myoblasts and oligodendroglia cells

a. Western blotting of total cell lysate from control and patient myoblasts indicates low EXOSC8 protein level and also show dramatic down-regulation of EXOSC3 in the patient cell line. Beta-actin and porin show even loading.

b. Immunostaining of control and patient myoblast with EXOSC8 (top row) and EXOSC3 (bottom row). Loss of both EXOSC8 and EXOSC3 was evident in the myoblasts derived from the patient.

c. Immunoblotting of EXOSC8 down-regulated human oligodendroglia cells. siRNA mediated downregulation of *EXOSC8* led to a dramatic loss of EXOSC3 suggesting functional correlation between the two proteins. Blotting for mitochondrial proteins such as NDUFB8 and COXI following EXOSC8 ablation revealed only minor changes in their protein levels.

d. Immunolabelling for both EXOSC8 and EXOSC3 also showed significantly lower signals (right) compared to non-targeting control samples (left).

Supplementary Figure 5

Whole Blots for Figure 5.

Figure 5 h. Exosc8

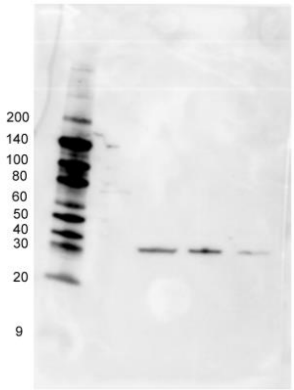

Figure 5 h. MBP

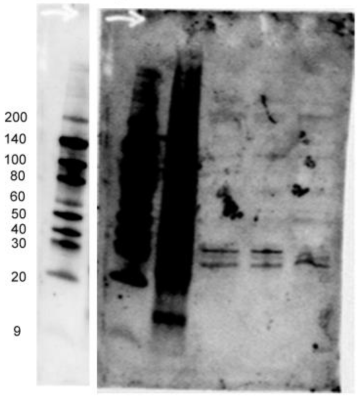

Figure 5 h. GAPDH

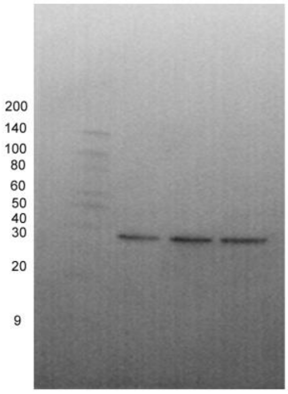

Figure 5 f. Exosc8

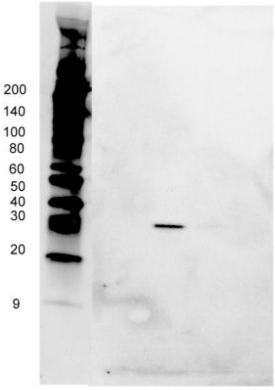

Figure 5 f. B-Actin

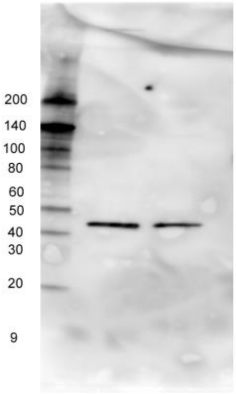

Figure 5 g. Exosc8

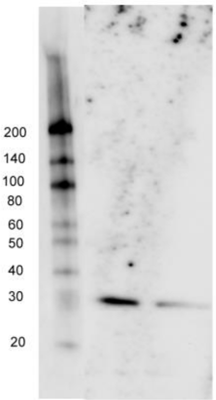

Figure 5 g. B-Actin

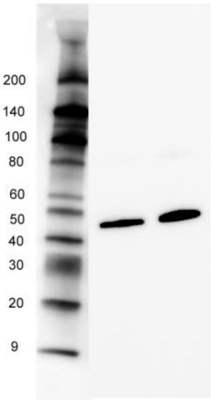

Whole Blots for Figure 3.

Figure 3 d Myo. Exosc8

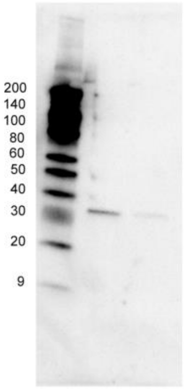

Figure 3 d Myo. Actin

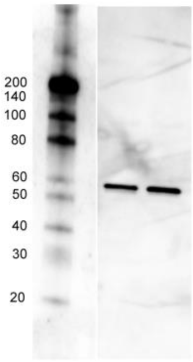

Figure 3 d Fibro.. Exosc8

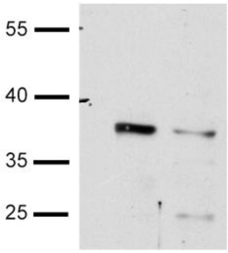

Figure 3d Fibro GAPDH

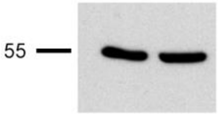

Patient	Onset/ Death	Clinical presentation							
		Neurological signs	Psychomo-tor ret.	Visual loss	Hearing loss	Respiratory problems	Other	Brain MRI	Other tests
P1 - V:20	2m/11m†	Severe muscle weakness and wasting, spastic tetraparesis	+	+	+	+	Facial dysmorphy, scoliosis, ing. hernia	No data	
P1 - V:10	2m/9m†	Severe muscle weakness and wasting, spastic tetraparesis	+	+	+	+	Tremor, irritability	Cortical atrophy, vermis hypoplasia, thin corpus callosum (2.5m)	Muscle biopsy: RC complex I+IV↓
P1 - V:9	4m/13m†	Muscle weakness, wasting, spasticity, facial dysmorphy	+	+	+	+	Inguinal hernia, axial hypotonia, no voice	Vermis hypoplasia (5m)	lactate↑
P1 - V:29	1m/14m†	Severe muscle weakness and wasting, spastic tetraparesis, contractures,	+	+	+	+	Tremor, axial hypotonia, brachy-cephalia, facial dysmorphy	Diffuse cortical and cerebellar atrophy (L>R), thin corpus callosum (11m)	Pathological BAEP
P1 - V:4	1.5m/18 m†	Severe tetraspasticity, muscle wasting, contractures, no spontaneous movements	+	+	+	+	Polyhydramnion dystrophy, tremor axial hypotonia	Thin corpus callosum, immature myelination (2m)	Metabolic acidosis, pathological BAEP, VEP
P1 - VI:3	12d/8m†	Severe spastic tetraparesis, reflexes↑	+	+	+	+	Tremor, feeding difficulties	No data	
P1 - V:2	2m/19m†	Severe spastic tetraparesis	+	+	+	+	Feeding difficulties, tremor, irritability	Some cortical atrophy (6m)	
P2 - II:7	1.5m/13 m†	Severe spastic tetraparesis, muscle wasting	+	+	+	+	Inguinal hernia, feeding difficulties, irritability, apnoe	No data	Transient ALT↑, GGT↑
P2 - II:10	2m/alive 9m	Severe spastic tetraparesis, muscle wasting	+	+	+	+	Feeding difficulties, irritability, apnoe	Thin corpus callosum, immature myelination (5m)	
P3 – II:1	6m/28m†	Severe muscle weakness and wasting, ankle contractures	+	-	-	+	Feeding difficulties	Vermis hypoplasia, mega cisterna magna	EMG: motor neuron lesion
P3 – II:3	4m/alive 5y	Severe muscle weakness, tongue fasciculations	+	-	-	+	Feeding difficulties	Vermis hypoplasia, mega cisterna magna	EMG: motor neuron lesion

Supplementary Table 1 Clinical presentation of 9 patients from 2 pedigrees (P1, P2) homozygous for c.815G>C, p.Ser272Thr and 2 patients from pedigree 3 (P3) homozygous for c.5C>T, p.Ala2Val in *EXOSC8*. Abbreviations: P: pedigree; m: month; ret.: retardation; BAEP: brainstem auditory evoked potentials, VEP: visual evoked potentials, ALT: alanine transaminase, GGT: gamma-glutamyl transferase

GENES INVOVED IN DEMYELINATION					
	Cont. siRNA 3d	Cont. siRNA 6d	EXOSC8 siRNA 3d	EXOSC8 siRNA 6d	AU rich elements
		Fold change ± SD	Fold change ± SD	Fold change ± SD	
MBP	1	0.92 ± 0.36	3.35 ± 0.55 *	6.58 ± 3.43*	Au Rich
MOBP	1	1.07 ± 0.51	3.74 ± 2.16 *	8.5 ± 5.02 *	Au Rich
AUH	1	0.83 ± 0.08	2.49 ± 0.49	1.27 ± 0.18	Au Rich
PLP1	1	0.74 ± 0.24	0.72 ± 0.32	0.17 ± 0.05	Non AU Rich
PMP22	1	1.15 ± 0.31	0.83 ± 0.05	0.9 ± 0.47	Non AU Rich
EIF2B2	1	0.99 ± 0.26	1.15 ± 0.27	1.27 ± 0.53	Non AU Rich
ABCD1	1	0.71 ± 0.07	1.95 ± 0.24	1.22 ± 0.35	Non AU Rich
ARSA	1	0.52 ± 0.52	0.91 ± 0.14	0.69 ± 0.16	Non AU Rich
**GENES INVOLVED IN ATAXIA**					
SACS	1	1.25±0.29	0.77±0.08	1.01±0.13	Au Rich
ATXN1	1	0.09±0.11	0.89±0.22	0.77±0.07	Au Rich
ATXN2	1	1.05±0.37	1.25±0.2	1.11±0.19	Au Rich
ATXN3	1	0.92±0.28	1.24±0.16	0.95±0.23	Non AU Rich
FXN	1	0.96±0.04	1.46±0.07	1.52±0.13	Non AU Rich
ADCK3	1	0.71±0.08	1.36±0.19	1.04±0.13	Non AU Rich
CACNA1A	1	0.53±0.06	0.59±0.08	0.46±0.06	Non AU Rich
**GENES INVOLVED IN MITOCHONDRIAL FUNCTION**					
COX7A2L	1	0.8±0.07	1.2±0.18	0.8±0.33	Au Rich
COX6A2	1	0.96±0.12	0.92±0.15	1.13±0.26	Au Rich
MRPS30	1	0.98±0.26	0.89±0.19	0.75±0.27	Au Rich
MRPL3	1	0.86±0.52	1.7±0.31	0.64±0.26	Au Rich
MTERF	1	0.63±0.11	1.17±0.31	0.82±0.23	Au Rich
TFBM1	1	1.85±0.35	1.38±0.54	1.76±0.22	Au Rich
TFAM	1	1.24±0.38	1.5±0.26	1.54±0.08	Au Rich
MTRF1	1	0.81±0.27	1.62±0.28	0.7±0.14	Au Rich
OPA1	1	1.08±0.17	1.43±0.12	1.39±0.2	Au Rich
SOD2	1	0.96±0.01	0.37±0.03	0.38±0.17	Au Rich
SURF1	1	0.94±0.4	0.96±0.35	0.83±0.3	Non AU Rich
RRM2B	1	0.76±0.18	1.72±0.43	1.12±0.27	Non AU Rich
EARS2	1	0.96±0.12	1.46±0.1	1.52±0.41	Non AU Rich
RARS2	1	0.81±0.37	1.85±0.49	0.94±0.24	Non AU Rich
DARS2	1	0.89±0.11	1.46±0.22	1.65±0.4	Non AU Rich
**GENES ASSOCIATED WITH MOTOR NEURON FUNCTION**					
SMN1	1	0.57±0.25	0.76±0.23	0.64±0.31	Au Rich
CRIMI	1	0.94±0.3	1.01±0.2	1.87±0.61	Au Rich
BICD2	1	0.47±0.21	0.55±0.3	0.62±0.26	Non AU Rich
IGHMBP2	1	0.17±0.15	0.53±0.08	1.34±0.28	Non AU Rich

Supplementary Table 2 AU-rich and non-AU-rich gene expression after *EXOSC8* down-regulation in myoblasts. Two AU-rich element containing genes, *MBP* and *MOBP* were significantly increased after 3 and 6 days of siRNA transfection in myoblasts. All data were normalized to day 3 control siRNA samples. Fold change and standard deviation are shown as the average of three independent measurements.

	Cont. siRNA 3d	EXOSC8 siRNA 3d	AU rich elements
		Fold change ± SD	
**GENES INVOVED IN DEMYELINATION**			
MBP	1	**6.74±1.89**	Au Rich
MOBP	1	**2.48±0.68**	Au Rich
PLP1	1	0.97±0.4	Non AU Rich
**GENES ASSOCIATED WITH MOTOR NEURON FUNCTION**			
SMN1	1	**2.72±0.1**	Au Rich
VRK1	1	1.1±0.3	Au Rich
CRIMI	1	1.49±0.42	Non AU Rich
BICD2	1	1.26±0.21	Non AU Rich
IGHMBP2	1	0.17±0.15	Non AU Rich

Supplementary Table 3 AU-rich and non-AU-rich element containing gene expression after *EXOSC8* down-regulation in fibroblasts. In addition to *MBP* and *MOBP* we detected increased *SMN1* in *EXOSC8* siRNA treated fibroblasts. All data were normalized to day 3 control siRNA samples. Fold change and standard deviation are shown as the average of three independent measurements.

**Human real-time PCR primers**		
	Forward 5´-3´	Reverse 5´-3´
“Myelin” primers		
MBP (ARE)	CTATAAATCGGCTCACAAGG	AGGCGGTTATATTAAGAAGC
MOBP (ARE)	ACTCCGAACACTTCAGCATACACT	GATCCAGTCCTCCTCTTTCTTCTG
AUH (ARE)	AGGTGGTGGTCTTGAACTGG	CACTGCTTTGGCTTCTTTGC
PMP22	TTCTCATCATCACCAAACGAA	ACTCATCACGCACAGACCAG
EIF2B2	ATCATTGGCACGAAGACCAT	TTGGGGAACTGTGGAGAAAG
ABCD1	AGGTTGGGAGGCTATGTGTG	ATGGAGAGCAGGGCAATG
PLP1	AGGCAGTCTCTGTGCTGATG	AGGTGGAAGGTCATTTGGAA
ARSA	CTGGACCTGCTGCCTACC	GGGTGAAGAAGTGAGCCTTG
“Ataxia” primers		
SACS (ARE)	GGAGCCATCAAAACTGGAAC	TCACCACCTTCAGCATCAAC
ATXN7 (ARE)	CCTACGCTGCCTCCCTACTT	ATCCTCTCTACGGTGCTGGA
ATXN2 (ARE)	TGAGGAACTTGAGGCTTTGG	ACTGGTTTGCCCTTGCTTC
ATXN3	ATTGCGAAGCTGACCAACTC	ATTCCTGAGCCATCATTTGC
FXN	TAGCAGAGGAAACGCTGGAC	CCTGGATGGAGAAGATAGCC
ADCK3	ACAAGCAGCACAAACAGACG	GACAGGAAAGGACTGGAACC
CACNA1A	TGCCTGATGATGACAAGACC	TCAAACTCCGTCCCAACTGT
“Spinal Muscular Atrophy / Motor Neuron” primers		
SMN1 (ARE)	ACCACACCTAAAAGAAAACCTGCT	CCGTCTTCTGACCAAATGGCAG
CRIM1 (ARE)	ACGCGATCACAATGGTTGTCGG	GGCATCAGTAAGGAAACCGAAGG
BICD2	TTCTCCTCGCTGCGTGCTATGT	AGCAGCGAGTTCAGCGTCTTCT
IGHMBP2	AGGAGGACGAACAGTCGAAAGG	GTTGTATGGCGAGACCACAGCA
“Mitochondrial” primers		
COX7A2L (ARE)	TTTAGTGGCTTCACGCAGAA	TGTGGAATCGGAGGTCAGTT
COX6A2 (ARE)	CGCCCCGAGTTCCGTCCCTA	GGGCAGAGGGTTCACGTGGC
MRPS30 (ARE)	ACGGTCACACCCAGTTTCA	GCTCCAGTCCAAGCAAAAAG
MRPL3 (ARE)	CAGGCACTCCTCTTTATGCTG	CTGTGGGTTTTCGTTTGACC
MTERF (ARE)	GGCTTTTTGGTGTGAAGTGTC	GGTCCTGCTCATTGGTAATCA
TFBM1 (ARE)	TTCAAAAGGAAGTGGCAGAGA	GGGCTGTATCAAGGGAGTGA
TFAM (ARE)	ATGTGGGGCGTGCTGAGT	TGCCAAGACAGATGAAAACC
MTRF1 (ARE)	GACAAGCGTCAGCAACAAAG	CCCTTCCCACCACATAAAAA
OPA1 (ARE)	CAATGATGTGGTCTTGTTTTGG	CTTGAGGTCTTCCGCCAGT
SOD2 (ARE)	GGGTTGGCTTGGTTTCAATA	GTAGTAAGCGTGCTCCCACA
SURF1	GCCTCATCTCCTCCTCAACTC	CCACTTCTCCCTCAATCTGG
RRM2B	CGCTGTTTCTATGGCTTTCA	CCACCACTCTTTCCCCAAA
EARS2	CTGGGGCAGCGGAGAATA	GGTGAGCAGAAACAGGGGTA
RARS2	TTCCCCAGAAGAAGATTGTG	CCAGTTCCCAGAAGACCAAA
DARS2	TTGCGTTCGTCTCACTTAGG	GCACCACAGATTCCACAGG
Control gene primers		
ACTB	CATCGAGCACGGCATCGTCA	TAGCACAGCCTGGATAGCAAC
TUBB	CTTCGGCCAGATCTTCAGAC	AGAGAGTGGGTCAGCTGGAA
GAPDH	AACAGCGACACCCATCCTC	CATACC AGGAAATGAGCTTGACAA

Supplementary Table 4 Human primer sequences used for RT-PCR.

**Zebrafish real-time PCR primers**		
	Forward 5´-3´	Reverse 5´-3´
Myelin-related primers		
mbp1	TTCTCCCTCTACCCCCAAAT	GGTGCCTTGACTCTCTCCAC
mbp2	GCCACTTTCTGTTTCCAAGG	CTCCCCCAGTCCAAATACCT
plp1 isoform A	GCCCGTGACAATCAAGACTA	GAGACAGCGACCACAAACTG
plp1 isoform B	CAGGGGTTGTTTGTGGAAAT	ACCGCAGAATAGCGTAGTTG
pmp22 isoform A	GCAGTGGAAATGGTGATGC	TGGCAGAGGAAGATGATGAA
pmp22 isoform B	CCAACTCTTCACGCTTCAGA	ATGATGGCTCCGCACATAAC
eif2b2	GGCACCACAGACAACATCTC	CAGAAAAGCCTCCACAGTCC
Control gene primers		
actb1	CGAGCTGTCTTCCCATCCA	TCACCAACGTAGCTGTCTTTCTG
ef1α	CTGGAGGCCAGCTCAAACAT	ATCAAGAAGAGTAGTACCGCTAGCATTAC
rpl13α	TCTGGAGGACTGTAAGAGGTATGC	AGACGCACAATCTTGAGAGCAG
exosc8 primers		
exosc8	TCAGCTGGTGCTTCCAAACAT	TATCCCACAGATCACGGTAGTG

Supplementary Table 5 Zebrafish primer sequences used for RT-PCR.

Supplementary Note 1

Mitochondrial studies in skeletal muscle biopsy of patient V:10 (pedigree 1)

Respiratory chain (RC) enzyme measurement

Muscle RC enzymes:	U/g NCP	U/U CS
NADH-CoQ-Oxidoreductase	**11.9↓** (15.8-42.8)	**0.13↓** (0.17-0.50)
Succ cytochrome c oxidoreductase	8.4 (6.0-25.0)	0.09 (0.08-0.45)
Cytochrome c Oxidase	**94↓** (112-351	**1.0↓** (1.1-5.0)
CS	92 (45-100)	

Blue-native gel electrophoresis (BN-PAGE) was normal in myoblasts of patient V:10.

Supplementary Note 2

Roma chromosome 13 (13q13.1) haplotype around the mutation detected in pedigrees 1 and 2 (c.815G>C, p.Ser272Thr)

The conserved 20-SNP haplotype, CAATGTTCTAACTAGACAGG, spanning 229522 nt between positions 37502597 (rs582091) to 37732119 (rs7327020) (hg19), was common in this population and occurred in the homozygous state in 7 out of 54 Bulgarian Roma controls. An additional group of 9/54 control subjects was homozygous for a smaller internal haplotype of 12 SNPs (123,530 nt), flanked by rs9547711 and rs7996290. However, only the affected pedigrees carried the c.815G>C, p.Ser272Thr variant on this relatively common roma haplotype, supporting pathogenicity.

Supplementary Note 3

Gene expression studies were performed for the following selected AU-rich element containing (ARE) genes and non ARE genes associated with similar clinical presentations

AU-rich element database (ARED) search: http://brp.kfshrc.edu.sa/ARED/

"MYELIN" ARE genes:

ACCESSION	UNIGENE TITLE	SOURCE mRNA	GENE	GENE ID	CHR
AK096859	Myelin basic protein	AK096859	MBP	4155	18
AK124766	Myelin-associated oligodendrocyte basic protein	AK124766	MOBP	4336	3
NM_001698	AU RNA binding protein/enoyl-Coenzyme A hydratase	NM_001698	AUH	549	9
NM_002677	Peripheral myelin protein 2	NM_002677	PMP2	5375	8

"MYELIN" non-ARE genes:

EIF2B2-vanishing white matter disease

PLP1-Pelizaeus-Merzbacher disease

ABCD1-adrenoleukodystrophy

ARSA-arylsulfatase A

PMP22-Charcot-Marie-Tooth disease type 1A

"ATAXIA" ARE genes:

ACCESSION	UNIGENE TITLE	SOURCE mRNA	GENE	GENE ID	CHR
NM_000332	Ataxin 1	NM_000332	ATXN1	6310	6
AK128613	Ataxin 2	NM_002973	ATXN2	6311	12
NM_014363	Spastic ataxia of Charlevoix-Saguenay (sacsin)	NM_014363	SACS	26278	13

"ATAXIA" non-ARE genes:

ATXN3-spinocerebellar ataxia type 3

FXN-Friedreich ataxia,

ADCK3-autosomal recessive cerebellar ataxia with coenyzme Q deficiency

CACNA1A-spinocerebellar ataxia type 6)

“SPINAL MUSCULAR AROPHY / MOTOR NEURON” ARE genes

Detail	Unigene	Gene	Locus	Human Mouse	Definition
Detail	Hs.19280	CRIM1	51232	MGI:1354756	cysteine-rich motor neuron 1
Detail	Hs.288986	SMN1	6606	MGI:109257	survival of motor neuron 1, telomeric

“SPINAL MUSCULAR AROPHY / MOTOR NEURON” non-ARE genes

BICD2-congenital distal spinal muscular atrophy

IGHMBP2-spinal muscular atrophy with respiratory distress type 1

“MITOCHONDRIAL” ARE genes

ACCESSION	UNIGENE TITLE	SOURCE mRNA	GENE	GENE ID	CHR
AF127788	Cytochrome c oxidase subunit VIIa polypeptide 2 like	AB007618	COX7A2L	9167	2
BC054007	Transcription factor B1, mitochondrial	BC054007	TFB1M	51106	6
BX538300	Mitochondrial ribosomal protein S30	BX538300	MRPS30	10884	5
NM_003201	Transcription factor A, mitochondrial	NM_003201	TFAM	7019	10
NM_004294	Mitochondrial translational release factor 1	NM_004294	MTRF1	9617	13
NM_005205	Cytochrome c oxidase subunit VIa polypeptide 2	NM_005205	COX6A2	1339	16
NM_006980	Mitochondrial transcription termination factor	NM_006980	MTERF	7978	7
NM_007208	Mitochondrial ribosomal protein L3	NM_007208	MRPL3	11222	3
NM_015560	Optic atrophy 1 (autosomal dominant)	NM_015560	OPA1	4976	3

“MITOCHONDRIAL” non-ARE genes:

SURF1-cytochrome c oxidase deficient Leigh syndrome

RRM2B-mitochondrial DNA depletion syndrome

EARS2-leukoencephalopathy with thalamus and brainstem involvement and high lactate

RARS2-pontocerebellar hypoplasia type 6, *DARS2*-leukoencephalopathy with brainstem and spinal cord involvement and elevated lactate)

AU-rich scores for zebrafish mRNAs: http://arescore.dkfz.de/arescore.pl.
